# Clinical Phenotyping and Treatment Response in Patients With Chronic Heart Failure

**DOI:** 10.1016/j.jacadv.2025.101972

**Published:** 2025-07-17

**Authors:** Daishiro Tatsuta, Motoki Nakao, Toshiyuki Nagai, Yoshifumi Mizuguchi, Isao Yokota, Taro Koya, Atsushi Tada, Suguru Ishizaka, Fusako George, Yoshiya Kato, Shogo Imagawa, Ko Motoi, Yusuke Tokuda, Masashige Takahashi, Junichi Matsumoto, Masaharu Machida, Hiroshi Okamoto, Takahiko Saito, Toshihisa Anzai, ELMSTAT-HF Investigators

**Affiliations:** a Department of Cardiovascular Medicine, Faculty of Medicine and Graduate School of Medicine, Hokkaido University, Sapporo, Japan; b Department of Biostatistics, Graduate School of Medicine, Hokkaido University, Sapporo, Japan; c Department of Cardiology, Kushiro City General Hospital, Kushiro, Hokkaido, Japan; d Department of Cardiology, National Hospital Organization Hakodate Medical Center, Hakodate, Hokkaido, Japan; e Department of Cardiology, Hokkaido Chuo Rosai Hospital, Iwamizawa, Hokkaido, Japan; f Division of Cardiology, Hakodate Municipal Hospital, Hakodate, Hokkaido, Japan; g Department of Cardiology, Japan Community Healthcare Organization Hokkaido Hospital, Sapporo, Japan; h Department of Cardiology, Keiwakai Ebetsu Hospital, Ebetsu, Japan; i Department of Cardiology, Tomakomai City Hospital, Tomakomai, Hokkaido, Japan; j Department of Cardiology, Aishin Memorial Hospital, Sapporo, Japan; k Department of Cardiology, Japan Red Cross Kitami Hospital, Kitami, Hokkaido, Japan

**Keywords:** heart failure, machine learning, medical therapy, phenotyping, prognosis

## Abstract

**Background:**

There are little data on clinically meaningful heart failure (HF) phenogroups, which are associated with treatment response across the wide spectrum of left ventricular (LV) ejection fraction.

**Objectives:**

The authors aimed to identify the phenotypes of patients with HF with different prognoses and responses to medical therapies.

**Methods:**

We examined consecutive 2,301 chronic HF patients from the ELMSTAT-HF (EpidemioLogical Multicenter Study for Tailored Treatment in Heart Failure) registry, a prospective multicenter cohort in which 2,317 patients were enrolled between January 2020 and September 2024. Latent class analysis was performed using 99 clinical features. The primary outcome was a composite of all-cause death and hospitalization for worsening HF.

**Results:**

The analysis subclassified the patients into 8 phenogroups: group 1, characterized by younger age with obesity; 2, less structural abnormality and comorbidity; 3, younger age with LV dilation; 4, LV hypertrophy; 5, older age with small LV and diastolic dysfunction; 6, ischemic cardiomyopathy; 7, advanced LV remodeling and ventricular arrhythmias; and 8, atrial myopathy. During a median follow-up of 597 (IQR: 302-932) days, the incidence of the primary outcome significantly differed between the phenogroups (*P* < 0.001). In phenogroup 5, patients taking beta-blockers or sodium-glucose cotransporter 2 inhibitors had a significantly higher rate of hospitalization for worsening HF (HR: 2.20; 95% CI: 1.04-4.68; HR: 4.27; 95% CI: 2.02-9.05, respectively).

**Conclusions:**

We identified 8 phenogroups with distinct clinical outcomes in patients with HF. This phenotyping provides appropriate risk stratification and may aid clinical decision-making in patients with HF.

Heart failure (HF) is increasingly common worldwide in parallel with the aging society despite advances in medical therapy and devices, leading to the social problem of an HF pandemic.^1^^,^^2^ HF is also a leading cause of hospitalization associated with poor clinical outcomes and high medical costs.^3^ Randomized clinical trials initially included patients with HF with reduced ejection fraction (HFrEF) to select high-risk study populations to enhance statistical power. Consequently, current guidelines recommend pharmacological and device therapies for HF based on the categories of left ventricular (LV) ejection fraction (LVEF).^4^, ^5^, ^6^

Nevertheless, the application of LVEF in clinical trials has led to an oversimplification of the scientific view of complex syndromes in HF. In fact, several studies showed that the risk of adverse events was unable to be well stratified by LVEF alone because of the multifactorial and heterogeneous nature of HF.^7^^,^^8^ A recent cohort study illustrated a U-shaped relationship between LVEF and the risk of adverse events, with the lowest risk noted at 60% to 65% of LVEF.^9^ In the subanalysis of STRONG-HF (The Safety, Tolerability and Efficacy of Rapid Optimization, Helped by NT-proBNP Testing, of Heart Failure Therapies) trial, which enrolled patients hospitalized for acute HF with any LVEF and not treated with full doses of renin-angiotensin system (RAS) blockers and beta-blockers, rapid uptitration of oral medications for HF and close follow-up reduced 180-day death and HF rehospitalization irrespective of LVEF.^10^ Furthermore, although the efficacy of sodium-glucose cotransporter 2 (SGLT2) inhibitors has been demonstrated for HF across all LVEF categories,^11^^,^^12^ these cohorts include patients with various underlying heart diseases and still have residual heterogeneity. Therefore, an emerging phenotypic classification of chronic HF with an integration of detailed clinical demographics, biomarkers, and parameters derived from imaging modalities would identify subgroups with different outcomes and treatment response patterns and is warranted to provide tailored strategies for individual patients with HF beyond the current classification based on LVEF.

One candidate approach is latent class analysis (LCA), which has received growing attention and has become widespread in recent epidemiological fields.^13^ LCA was initially developed to isolate specific phenotypes of individuals with similar characteristics based on probability calculations; it offers a strategy to identify subgroups of patients with HF who are more susceptible to adverse clinical events. Recent studies have demonstrated the feasibility of LCA and similar approaches for phenomapping in acute and chronic HF with preserved ejection fraction (HFpEF) with heterogeneous pathophysiologies, mainly from data from randomized clinical trials.^14^^,^^15^ In addition, a study on chronic HF with a wide range of LVEF categories using data from a registry was conducted between 2006 and 2010.^16^ However, there is a paucity of findings with clinically meaningful phenogroups, which are associated with treatment response to guideline-derived medical therapy across the wide spectrum of LVEF based on analyses of real-world registries in the era of current guidelines.

Accordingly, we sought to identify the phenogroups of patients with chronic HF with different prognoses and responses to medical therapies using data from a multicenter prospective registry.

## Methods

### Study design and data sources

Data from the ELMSTAT-HF (EpidemioLogical Multicenter Study for Tailored Treatment in Heart Failure) registry, collected between January 2020 and September 2024, were analyzed. Details of the ELMSTAT-HF registry have been described elsewhere.^17^ Briefly, the ELMSTAT-HF study includes 28 centers in Japan and is a prospective, observational, ongoing cohort study of patients with chronic HF aged ≥20 years. This study aims to develop tailored treatment strategies for patients with chronic HF by collecting information, including blood biomarkers, genomes, and metabolomes, at enrollment.^17^ Clinical parameters, including laboratory and echocardiographic data, were collected either at the time of outpatient visits when HF was well-compensated or at discharge after appropriate inpatient management. The current list of the study centers and investigators is provided in Supplemental Appendix 1. Chronic HF was diagnosed according to the current HF clinical guidelines.^4^^,^^5^ Patients were enrolled as inpatients or outpatients. The exclusion criteria were acute coronary syndrome, acute myocarditis, sepsis, the expected need for open-heart surgery within 4 weeks, postheart transplantation, and LV assist device implantation, waiting for heart transplantation. Follow-up was performed by dedicated coordinators and investigators at 9, 18, and 24 months after enrollment. Follow-ups were repeated every 12 months thereafter by direct contact with patients or their physicians at the hospital or outpatient clinic, telephone interviews with patients or, if deceased, with family members, and by mail. This study was approved by the Institutional Review Boards of each center and registered under the Japanese UMIN Clinical Trials Registration (UMIN000043390). The study conformed to the principles outlined in the Declaration of Helsinki. All the patients provided written informed consent to participate in the study.

### Study population

**Figure 1:**
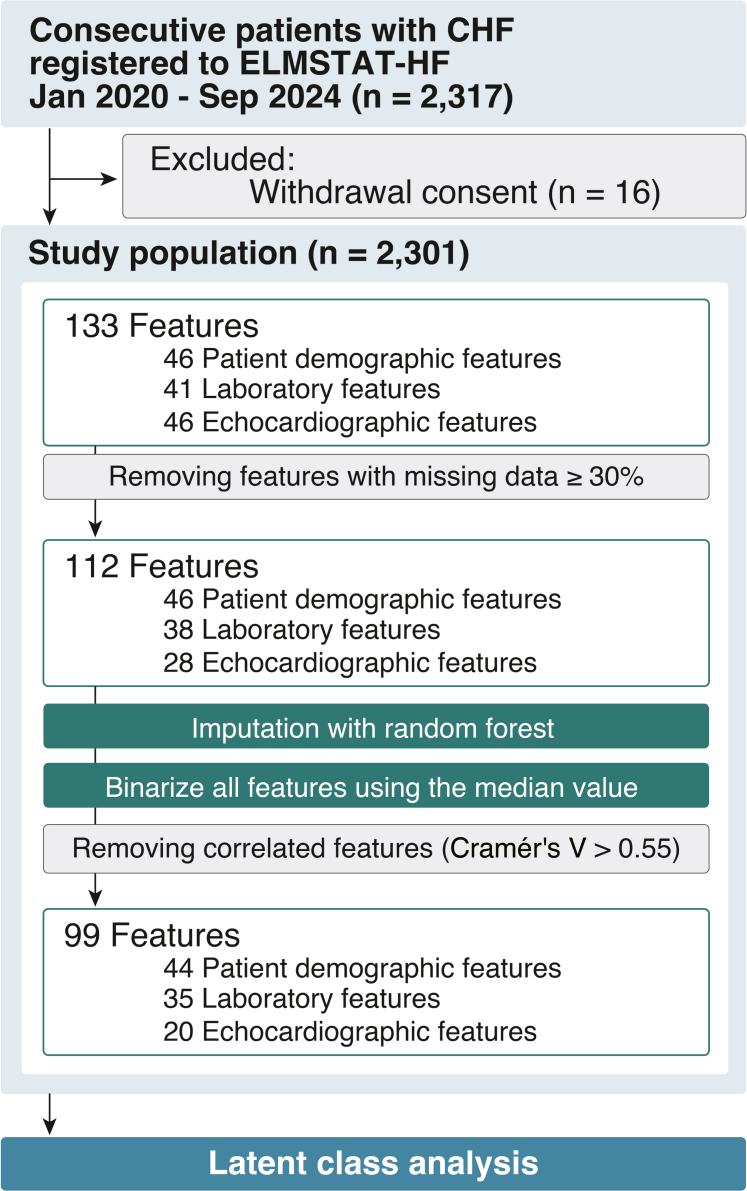
Flow Diagram of the Study CHF = chronic heart failure; ELMSTAT-HF = EpidemioLogical Multicenter Study for Tailored Treatment in Heart Failure.

Of the 2,317 patients enrolled in the ELMSTAT-HF registry, we excluded those who withdrew research consent (n = 16). Ultimately, 2,301 patients were included in this study (Figure 1).

### Latent class analysis

A total of 133 features on enrollment were considered as primary candidates for LCA. The variables included patient demographic (46 features), laboratory (41 features), and echocardiographic (46 features) data. Twenty-one features were excluded in the present analyses because over 30% of their data were missing. Details of the missing data for all features are shown in Supplemental Table 1. For variables with missing data, random forest imputation using the “missForest” package was performed prior to variable selection, which allows flexible and accurate imputation of both continuous and categorical variables without assuming linearity.^18^ After binarizing all features using the median value, we removed correlated features with Cramér’s V >0.55. Finally, 99 binarized variables were used in the LCA. The correlations between the selected variables are shown in Supplemental Figure 1. The LCA was performed using the “poLCA” package in R software (version 4.4.2; R Foundation for Statistical Computing) to attempt using 2 to 15 clusters to identify the optimal number of clusters. The optimal number of clusters was determined by the optimization of the Bayesian information criterion (BIC). The model with the lowest BIC value provided the optimal number of clusters. In this analysis, 8 clusters had the lowest BIC values (Supplemental Figure 2, Supplemental Table 2). The importance of each feature value between the phenogroups was calculated using Cramér's V. After identifying the number of optimal clusters, we assessed the differences in patient demographics, laboratory data, echocardiographic data, and medications. For internal validation, 1,000 bootstrap samplings were conducted.^19^ In each resampled data set, random forest imputation and binarization were conducted, similar to that in the derivation cohort, and LCA was applied using the same 99 features. The optimal number of clusters was reassessed by comparing the BIC values in 10 bootstrap data sets randomly generated from the original cohort (Supplemental Figure 3). In 8 out of the 10 data sets, the 8-cluster solution showed the lowest BIC value, and in all data sets, BIC increased when moving from 8 to 9 clusters. These findings support 8 as the optimal number of clusters. To align the phenogroup labels across bootstrap iterations, we used the Hungarian algorithm.

### Clinical outcomes

The primary outcome of this study was a composite of all-cause death and hospitalization due to worsening HF. The secondary outcomes were the individual components of the primary outcome and the differences in the primary outcome according to the use of RAS blockers, beta-blockers, mineralocorticoid receptor antagonists, or SGLT2 inhibitors among the phenogroups. The treating physicians at each participating hospital identified HF rehospitalizations according to the standard definitions.

### Statistical analysis

Continuous variables were presented as mean ± SD when normally distributed, and as median (IQR) when non-normally distributed. Differences between phenogroups were compared through one-way analysis of variance or the Kruskal-Wallis test for continuous variables and the chi-squared test or Fisher exact test for categorical variables, as appropriate. A Sankey diagram was constructed to visually depict the transition frequencies between the classification based on LVEF and the 8 derived phenogroups. The cumulative incidence of primary and secondary outcomes was estimated using the Kaplan-Meier survival function and compared among the phenogroups using the log-rank test. Cox proportional hazards analyses were performed to evaluate the risk of primary and secondary outcomes among the phenogroups. The proportional hazards assumption was assessed using log-minus-log survival plots for each outcome. In addition, subgroup analyses were performed within each phenogroup to assess the association between prescription of medications and outcomes. In the multivariable models, N-terminal pro-brain natriuretic peptide and the Meta-Analysis Global Group in Chronic Heart Failure risk score were used as covariates, based on previous studies.^20^^,^^21^ The LCA and other statistical analyses were performed using STATA MP 16 (StataCorp) and R software (version 4.4.2, R Foundation for Statistical Computing), with partial script development supported by ChatGPT-4 (OpenAI). A *P* value <0.05 was considered to be statistically significant.

## Results

### Phenogroup characteristics

**Figure 2:**
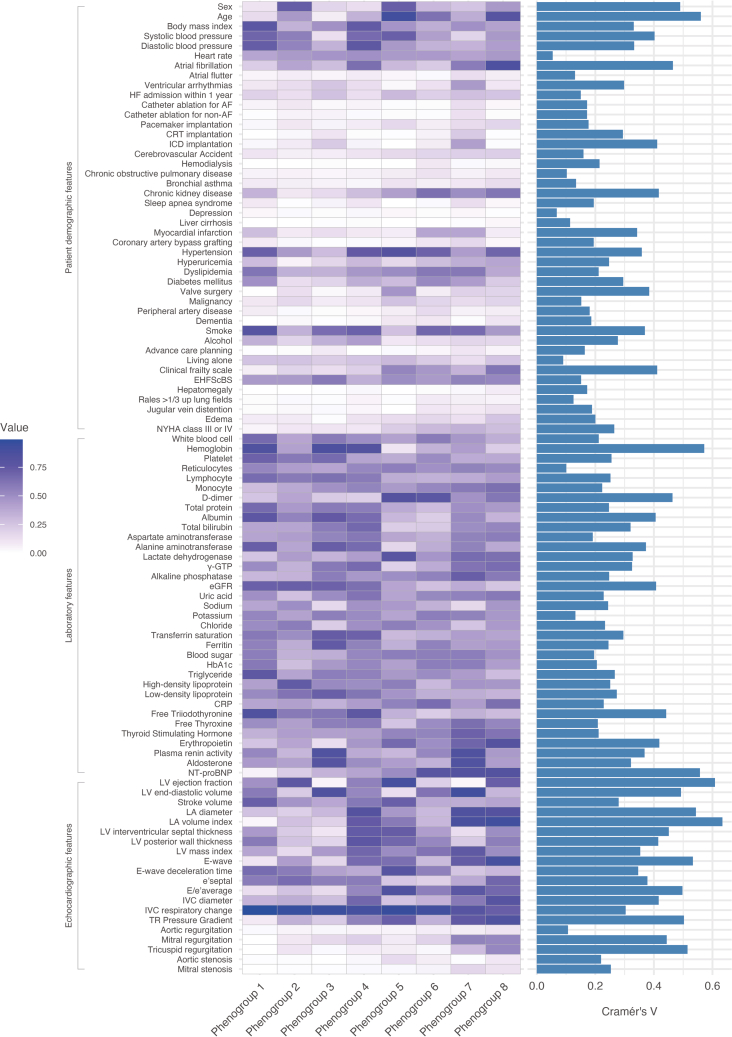
Expression Patterns and Feature Importance Across Phenogroups Lower levels of the variables are represented by lighter colors, while darker colors indicate higher levels. The importance of each variable in defining the phenogroups is illustrated using bar plots. AF = atrial fibrillation; CRP = C-reactive protein; CRT = cardiac resynchronization therapy; eGFR = estimated glomerular filtration rate; EHFScBS = European Heart Failure Self-care Behaviour Scale; HbA1c = hemoglobin A1c; HF = heart failure; ICD = implantable cardioverter-defibrillator; IVC = inferior vena cava; LA = left atrium; LV = left ventricle; NT-proBNP = N-terminal pro B-type natriuretic peptide; TR = tricuspid regurgitation; γ-GTP = gamma-glutamyl transpeptidase.

The patients were subclassified into 8 phenogroups as follows: phenogroup 1 (n = 245/2,301, 10.6%), 2 (n = 329/2,301, 14.3%), 3 (n = 251/2,301, 10.9%), 4 (n = 336/2,301, 14.6%), 5 (n = 256/2,301, 11.1%), 6 (n = 361/2,301, 15.7%), 7 (n = 177/2,301, 7.7%), and 8 (n = 346/2,301, 15.0%) and were labeled based on characteristics such as “younger age with obesity,” “less structural abnormality and comorbidity,” “younger age with LV dilation,” “LV hypertrophy,” “older age with small LV and diastolic dysfunction,” “ischemic cardiomyopathy,” “advanced LV remodeling and ventricular arrhythmias,” and “atrial myopathy,” respectively. The representative demographic, laboratory, and echocardiographic data are presented in Table 1. All 99 features used in the LCA and expression patterns across the clusters and importance of each feature are depicted in the heatmap and bar plot in Figure 2.

**Table 1:** Baseline Characteristics

	All Patients (N = 2,301)	Phenogroup 1	Phenogroup 2	Phenogroup 3	Phenogroup 4	Phenogroup 5	Phenogroup 6	Phenogroup 7	Phenogroup 8	*P* Value
		Younger Age With Obesity (n = 245)	Less Structural Abnormality and Comorbidity (n = 329)	Younger Age With LV Dilation (n = 251)	LV Hypertrophy (n = 336)	Older Age With Small LV and Diastolic Dysfunction (n = 256)	ICM (n = 361)	Advanced LV Remodeling and VAs (n = 177)	Atrial Myopathy (n = 346)	
Patient demographic data										
Age, y	74.0 (65.0-82.0)	64.0 (54.0-71.0)	73.0 (65.0-80.0)	58.0 (49.0-68.0)	71.0 (63.0-77.0)	84.0 (80.0-88.0)	77.0 (71.0-83.0)	71.0 (62.0-77.0)	82.0 (77.0-86.0)	<0.001
Male	1,442 (62.7)	216 (88.2)	84 (25.5)	208 (82.9)	293 (87.2)	70 (27.3)	247 (68.4)	132 (74.6)	192 (55.5)	<0.001
BMI, kg/m^2^	22.9 (20.5-25.8)	25.7 (23.4-28.3)	21.5 (19.6-23.7)	22.7 (20.6-26.0)	25.3 (22.9-28.4)	22.7 (20.1-25.0)	21.8 (19.4-24.0)	22.9 (20.8-25.8)	22.1 (19.7-24.5)	<0.001
Systolic blood pressure, mm Hg	117.6 ± 19.7	125.1 ± 15.8	121.5 ± 19.6	102.8 ± 12.9	125.7 ± 18.4	127.2 ± 19.4	113.8 ± 17.7	102.8 ± 18.8	116.2 ± 18.2	<0.001
Heart rate, beats/min	70.3 ± 13.6	68.6 ± 12.7	71.3 ± 13.9	70.4 ± 12.7	71.7 ± 15.4	70.1 ± 13.8	69.5 ± 12.2	69.1 ± 11.4	70.7 ± 14.6	0.11
HF admission within 1 y	487 (21.4)	46 (19.1)	42 (12.8)	69 (27.7)	47 (14.0)	79 (31.1)	71 (19.8)	47 (27.0)	86 (25.7)	<0.001
Smoking	1,276 (56.4)	190 (80.5)	113 (34.7)	162 (65.3)	233 (70.8)	63 (24.9)	242 (68.8)	116 (66.7)	157 (45.8)	<0.001
Alcohol	534 (23.6)	78 (32.9)	62 (19.1)	89 (36.2)	145 (43.9)	23 (9.1)	48 (13.6)	30 (17.2)	59 (17.4)	<0.001
NYHA class III or IV	422 (18.6)	11 (4.5)	33 (10.1)	27 (10.8)	30 (9.1)	57 (22.4)	111 (31.2)	59 (33.9)	94 (27.6)	<0.001
Clinical Frailty Scale										<0.001
Low (1-3)	1,480 (66.5)	224 (93.7)	262 (81.6)	203 (84.9)	246 (78.1)	114 (45.8)	187 (53.3)	114 (66.3)	130 (38.5)	
High (4-9)	744 (33.5)	15 (6.3)	59 (18.4)	36 (15.1)	69 (21.9)	135 (54.2)	164 (46.7)	58 (33.7)	208 (61.5)	
Past history										
Atrial fibrillation	1,033 (45.1)	52 (21.4)	132 (40.2)	70 (28.0)	213 (63.6)	78 (30.6)	82 (22.8)	105 (60.3)	301 (87.5)	<0.001
Ventricular arrhythmia	339 (14.9)	23 (9.5)	46 (14.1)	63 (25.6)	45 (13.5)	6 (2.4)	41 (11.5)	81 (46.0)	34 (9.9)	<0.001
CRT	108 (4.7)	0 (0.0)	9 (2.7)	27 (10.8)	4 (1.2)	3 (1.2)	21 (5.8)	41 (23.3)	3 (0.9)	<0.001
ICD	213 (9.3)	8 (3.3)	27 (8.2)	60 (24.0)	12 (3.6)	0 (0.0)	24 (6.7)	77 (43.8)	5 (1.5)	<0.001
Stroke	317 (13.9)	25 (10.4)	22 (6.7)	17 (6.8)	39 (11.7)	38 (14.9)	65 (18.1)	36 (20.5)	75 (21.8)	<0.001
COPD	160 (7.0)	13 (5.4)	18 (5.5)	9 (3.6)	18 (5.4)	18 (7.1)	43 (11.9)	10 (5.7)	31 (9.0)	0.001
Chronic kidney disease	883 (38.6)	83 (34.3)	28 (8.6)	37 (14.8)	94 (28.2)	108 (42.4)	234 (65.0)	87 (49.4)	212 (61.8)	<0.001
Hypertension	1,417 (62.1)	173 (72.7)	148 (45.3)	70 (28.0)	252 (75.4)	210 (82.4)	251 (70.1)	74 (42.3)	239 (69.7)	<0.001
Dyslipidemia	1,087 (47.6)	148 (61.7)	115 (35.2)	88 (35.2)	153 (45.8)	129 (50.6)	215 (59.7)	106 (60.9)	133 (38.7)	<0.001
Diabetes mellitus	745 (32.8)	119 (49.8)	42 (13.0)	48 (19.2)	118 (35.5)	74 (29.2)	186 (52.0)	78 (44.6)	80 (23.4)	<0.001
Myocardial infarction	416 (18.2)	68 (28.2)	27 (8.2)	38 (15.2)	29 (8.7)	19 (7.5)	139 (38.5)	72 (40.9)	24 (7.0)	<0.001
Malignancy	355 (15.6)	22 (9.1)	55 (16.8)	17 (6.8)	39 (11.7)	67 (26.4)	63 (17.5)	27 (15.4)	65 (19.0)	<0.001
Valve surgery	316 (13.9)	1 (0.4)	42 (12.8)	6 (2.4)	26 (7.8)	119 (46.9)	23 (6.4)	34 (19.3)	65 (19.0)	<0.001
CABG	124 (5.4)	20 (8.3)	10 (3.0)	2 (0.8)	7 (2.1)	9 (3.5)	34 (9.4)	30 (17.0)	12 (3.5)	<0.001
PCI	550 (24.1)	76 (31.5)	43 (13.1)	43 (17.3)	62 (18.6)	57 (22.4)	152 (42.2)	66 (37.7)	51 (14.9)	<0.001
Etiology										<0.001
ICM	543 (23.6)	97 (39.6)	39 (11.9)	49 (19.5)	49 (14.6)	25 (9.8)	185 (51.2)	73 (41.2)	26 (7.5)	
NICM	633 (27.5)	88 (35.9)	76 (23.1)	137 (54.6)	106 (31.5)	20 (7.8)	82 (22.7)	68 (38.4)	56 (16.2)	
Valve disease	431 (18.7)	4 (1.6)	53 (16.1)	4 (1.6)	41 (12.2)	158 (61.7)	36 (10.0)	13 (7.3)	122 (35.3)	
Others	694 (30.2)	56 (22.9)	161 (48.9)	61 (24.3)	140 (41.7)	53 (20.7)	58 (16.1)	23 (13.0)	142 (41.0)	
Laboratory data										
Hemoglobin, g/dL	12.7 ± 2.1	14.2 ± 1.7	12.3 ± 1.5	14.4 ± 1.6	14.2 ± 1.7	10.9 ± 1.5	11.9 ± 1.8	12.6 ± 1.9	11.4 ± 1.7	<0.001
D-dimer, μg/mL	0.8 (0.5-1.8)	0.6 (0.5-0.9)	0.7 (0.5-1.3)	0.6 (0.3-0.8)	0.6 (0.5-0.8)	1.7 (1.1-3.2)	1.7 (0.9-3.4)	0.8 (0.5-1.8)	1.1 (0.6-2.5)	<0.001
Albumin, g/dL	3.8 ± 0.5	4.1 ± 0.4	3.9 ± 0.4	4.1 ± 0.4	4.0 ± 0.4	3.6 ± 0.5	3.5 ± 0.5	3.9 ± 0.4	3.6 ± 0.5	<0.001
γ-GTP, U/L	30.0 (19.0-54.0)	31.0 (21.0-55.0)	22.0 (15.0-37.0)	37.0 (24.0-64.0)	39.0 (25.0-65.0)	19.0 (14.0-28.0)	24.5 (17.0-42.0)	40.0 (24.0-72.0)	42.0 (24.0-68.0)	<0.001
eGFR, mL/min/1.73 m^2^	52.0 ± 20.4	59.4 ± 17.9	61.2 ± 17.4	62.3 ± 22.1	57.2 ± 17.1	47.6 ± 18.2	41.3 ± 20.1	46.5 ± 19.9	42.8 ± 17.0	<0.001
Sodium, mEq/L	139.4 ± 3.3	140.0 ± 2.4	140.0 ± 3.0	138.4 ± 2.8	140.0 ± 3.3	139.8 ± 3.3	138.9 ± 3.7	137.4 ± 3.2	139.8 ± 3.4	<0.001
Potassium, mEq/L	4.2 ± 0.5	4.2 ± 0.4	4.1 ± 0.4	4.2 ± 0.4	4.2 ± 0.4	4.1 ± 0.5	4.3 ± 0.5	4.3 ± 0.6	4.1 ± 0.5	<0.001
Chloride, mEq/L	103.7 ± 3.7	104.2 ± 2.9	104.7 ± 3.2	102.4 ± 2.9	104.0 ± 3.2	104.8 ± 3.6	103.7 ± 4.0	101.6 ± 4.4	103.4 ± 4.1	<0.001
Ferritin, ng/mL	101.0 (44.5-207.0)	122.5 (55.3-235.0)	76.7 (31.5-150.5)	193.9 (101.0-321.2)	115.4 (48.0-216.6)	69.0 (36.0-142.0)	112.0 (46.0-262.0)	80.5 (34.0-179.9)	83.2 (37.2-165.0)	<0.001
Transferrin saturation, %	27.3 ± 13.5	29.3 ± 12.2	26.9 ± 12.3	35.3 ± 14.9	32.1 ± 14.0	22.4 ± 10.5	23.9 ± 12.0	25.0 ± 10.0	24.0 ± 14.5	<0.001
hs-Troponin T, ng/L	17.0 (8.0-37.0)	10.0 (5.0-19.0)	8.0 (5.0-14.0)	9.0 (5.0-18.0)	17.0 (10.0-27.0)	20.0 (9.0-40.0)	35.0 (19.0-64.0)	22.0 (11.0-40.0)	31.0 (16.0-58.0)	<0.001
Free Triiodothyronine, pg/mL	2.5 ± 0.6	2.9 ± 0.6	2.6 ± 0.6	2.6 ± 0.5	2.7 ± 0.5	2.3 ± 0.5	2.2 ± 0.6	2.4 ± 0.7	2.3 ± 0.5	<0.001
Erythropoietin, U/L	13.2 (8.7-21.6)	10.4 (7.4-14.8)	11.5 (8.1-17.0)	7.9 (5.3-11.8)	13.1 (9.0-19.6)	17.0 (11.0-27.9)	12.7 (8.4-20.4)	17.6 (11.7-28.1)	22.9 (14.6-36.1)	<0.001
NT-proBNP, pg/mL	820 (372-1,875)	284 (126-512)	406 (218-809)	538 (267-1,011)	699 (392-1,315)	802 (412-1,906)	1,984 (972-3,608)	1,578 (1,078-2,805)	1,828 (968-3,524)	<0.001
CRP, mg/dL	0.1 (0.1-0.4)	0.1 (0.0-0.2)	0.1 (0.0-0.2)	0.1 (0.0-0.2)	0.1 (0.1-0.3)	0.2 (0.1-0.8)	0.3 (0.1-0.9)	0.1 (0.0-0.3)	0.2 (0.1-0.6)	<0.001
Iron deficiency	1,231 (53.5)	113 (46.1)	208 (63.2)	67 (26.7)	158 (47.0)	185 (72.3)	186 (51.5)	106 (59.9)	208 (60.1)	<0.001
Anemia	1,098 (47.7)	40 (16.3)	146 (44.4)	33 (13.1)	66 (19.6)	212 (82.8)	247 (68.4)	102 (57.6)	252 (72.8)	<0.001
Echocardiographic data										
LVEF, %	48.8 ± 15.7	49.8 ± 12.8	57.4 ± 12.3	33.7 ± 9.6	51.3 ± 14.4	63.4 ± 10.1	40.3 ± 12.0	31.0 ± 8.8	55.2 ± 12.5	<0.001
LV end-diastolic volume, mL	108.9 ± 54.3	111.0 ± 40.6	78.5 ± 27.4	147.0 ± 56.4	111.8 ± 50.1	73.1 ± 24.1	119.0 ± 56.4	175.0 ± 63.4	88.3 ± 38.1	<0.001
LV end-systolic volume, mL	61.9 ± 47.2	58.4 ± 32.6	35.7 ± 20.8	100.9 ± 50.3	58.9 ± 41.7	28.6 ± 16.6	75.6 ± 47.2	124.1 ± 55.0	42.0 ± 28.5	<0.001
Stroke volume, mL	55.2 ± 17.9	61.4 ± 17.0	53.7 ± 17.6	52.8 ± 15.7	58.1 ± 20.1	62.7 ± 17.1	51.2 ± 17.2	50.4 ± 15.3	53.0 ± 17.6	<0.001
IVS, mm	10.3 ± 2.6	10.5 ± 2.2	9.4 ± 2.5	8.5 ± 1.7	11.9 ± 2.7	11.7 ± 2.3	10.1 ± 2.2	8.6 ± 2.2	10.7 ± 2.6	<0.001
LV mass index, g/m^2^	120.1 ± 37.6	112.5 ± 33.8	94.2 ± 25.2	114.4 ± 31.3	135.0 ± 41.1	118.4 ± 32.6	127.6 ± 35.9	139.1 ± 37.2	122.7 ± 40.2	<0.001
LAVI, mL/m^2^	54.0 ± 33.8	33.4 ± 9.1	41.7 ± 17.3	38.8 ± 15.5	59.4 ± 22.1	53.5 ± 18.1	43.3 ± 13.6	71.7 ± 38.7	85.1 ± 58.0	<0.001
RA diameter, mm	41.3 ± 15.5	37.3 ± 6.4	37.4 ± 7.3	39.9 ± 7.4	43.2 ± 8.6	38.3 ± 6.9	39.1 ± 33.4	44.3 ± 8.5	48.1 ± 11.0	<0.001
TRPG, mm Hg	25.6 ± 10.1	18.1 ± 5.2	22.5 ± 7.8	21.1 ± 6.0	24.9 ± 8.6	27.5 ± 7.3	22.6 ± 9.0	32.8 ± 12.5	32.2 ± 11.3	<0.001
E/e’ average	14.2 ± 6.9	9.8 ± 3.5	11.7 ± 5.0	9.9 ± 3.4	14.3 ± 5.7	18.3 ± 6.8	15.0 ± 6.2	19.0 ± 10.6	16.4 ± 7.4	<0.001
Aortic regurgitation	131 (6.0)	5 (2.2)	17 (5.4)	9 (3.8)	16 (4.8)	12 (5.3)	24 (6.8)	11 (6.4)	37 (11.0)	<0.001
Mitral regurgitation	502 (22.4)	0 (0.0)	29 (9.1)	36 (14.9)	65 (19.6)	19 (7.6)	74 (20.8)	97 (55.7)	182 (53.4)	<0.001
Tricuspid regurgitation	328 (14.6)	1 (0.4)	34 (10.6)	6 (2.5)	23 (6.9)	17 (6.8)	10 (2.8)	51 (29.3)	186 (54.2)	<0.001
Aortic stenosis	117 (5.5)	2 (0.9)	7 (2.4)	0 (0.0)	9 (2.8)	39 (17.5)	24 (7.0)	3 (1.7)	33 (10.1)	<0.001

Values are mean ± SD, median (IQR) or n (%).

BMI = body mass index; CABG = coronary artery bypass grafting; COPD = chronic obstructive pulmonary disease; CRP = C-reactive protein; CRT = cardiac resynchronization therapy; eGFR = estimated glomerular filtration rate; HF = heart failure; hs-Troponin T = high-sensitivity; ICD = implantable cardioverter-defibrillator; ICM = ischemic cardiomyopathy; IVS = interventricular septum; LAVI = left atrial volume index; LV = left ventricle; LVEF = left ventricular ejection fraction; NICM = nonischemic cardiomyopathy; NT-proBNP = N-terminal pro-brain natriuretic peptide; PCI = percutaneous coronary intervention; RA = right atrial; TRPG = tricuspid regurgitation pressure gradient; VA = ventricular arrhythmia.; γ-GTP = gamma-glutamyl transpeptidase.

Phenogroup 1 was comprised of small left atrium (LA), little congestion, younger age, and high body mass index (BMI) (Supplemental Figure 4). Patients in this group had the highest prevalence of dyslipidemia and smoking. Phenogroup 2 was characterized by a higher proportion of female, fewer structural abnormalities in echocardiographic features, and even fewer comorbidities (Supplemental Figure 5). Phenogroup 3 was characterized by a low LVEF, a greater LV volume, and a younger age profile. In addition, this phenogroup had elevated plasma renin activity and aldosterone levels (Supplemental Figure 6). Phenogroup 4 exhibited higher LV interventricular septal wall and posterior wall thickness, along with an enlarged LA. This phenogroup also had higher BMI and blood pressure (Supplemental Figure 7). Phenogroup 5 predominantly consisted of very elderly female, many of whom had a history of valvular surgery. Additionally, they exhibited a high LVEF, a small LV size, and an elevated average E/e’ (Supplemental Figure 8). Among all phenogroups, this phenogroup had the highest prevalence of a Clinical Frailty Scale score of 4 or higher, as well as the lowest hemoglobin levels and transferrin saturation. Phenogroup 6 exhibited a low LVEF and elevated D-dimer levels. This phenogroup frequently had a history of chronic kidney disease, myocardial infarction, dialysis, and peripheral artery disease (Supplemental Figure 9). Phenogroup 7 had a high prevalence of implantable cardioverter-defibrillator and cardiac resynchronization therapy implantation, as well as a history of ventricular arrhythmias. In this phenogroup, LVEF was low, and both the LV and LA were enlarged with mitral regurgitation (Supplemental Figure 10). Phenogroup 8 was characterized by a markedly enlarged LA, an extremely high prevalence of atrial fibrillation and mitral regurgitation. In addition, this phenogroup had preserved LVEF without dilated LV (Supplemental Figure 11), and the largest right atrial diameter compared to the other phenogroups (Table 1).

In the 8 phenogroups, key variables included LA and LV size and LVEF under echocardiographic features; age, sex, history of atrial fibrillation, chronic kidney disease, implantable cardioverter-defibrillator implantation, and degree of frailty under patient demographics; and N-terminal pro-B-type natriuretic peptide levels, hemoglobin levels, D-dimer, estimated glomerular filtration rate, albumin levels, and free triiodothyronine under laboratory features (Figure 1). In contrast, physical findings such as hepatomegaly and jugular venous distension, as well as the presence of chronic obstructive pulmonary disease were not key factors for phenogrouping in our patient cohort. Most HFpEF cases belonged to phenogroups 2, 4, 5, and 8, whereas most HFrEF cases belonged to phenogroups 3, 6, and 7 (Supplemental Figure 12).

### Association of phenogroups with clinical outcomes and treatments

**Figure 3:**
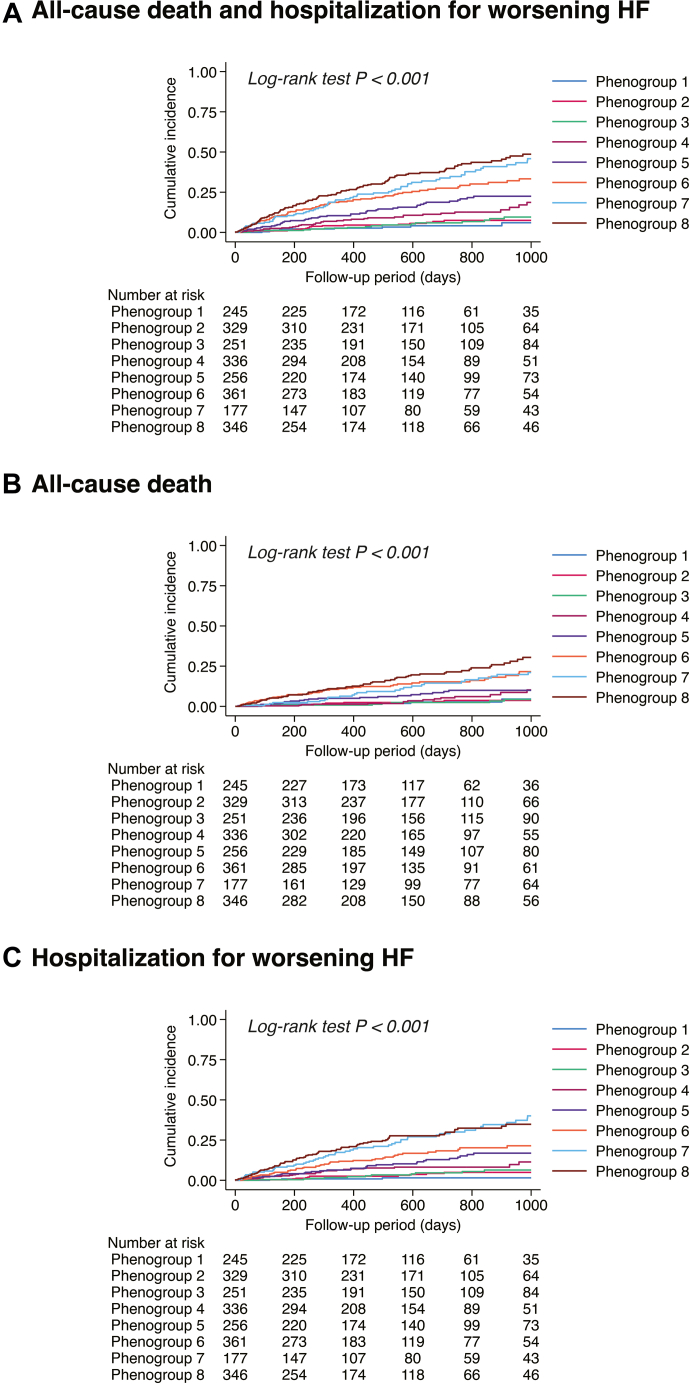
Cumulative Incidence of Clinical Outcomes Among the 8 Phenogroups (A) All-cause death and HF hospitalization. (B) All-cause death. (C) HF hospitalization. Abbreviations as in Figure 2.

During the median follow-up period of 597 (IQR: 302-932) days, the primary outcome occurred in 420/2,301 patients (18.3%). The log-minus-log survival plots demonstrated approximately parallel curves across phenogroups, supporting the validity of the proportional hazards assumption (Supplemental Figure 13). The Kaplan-Meier analysis showed a significant difference in each clinical outcome across the phenogroups (Figure 3, Supplemental Figures 14 to 16, Supplemental Table 3).

**Figure 4:**
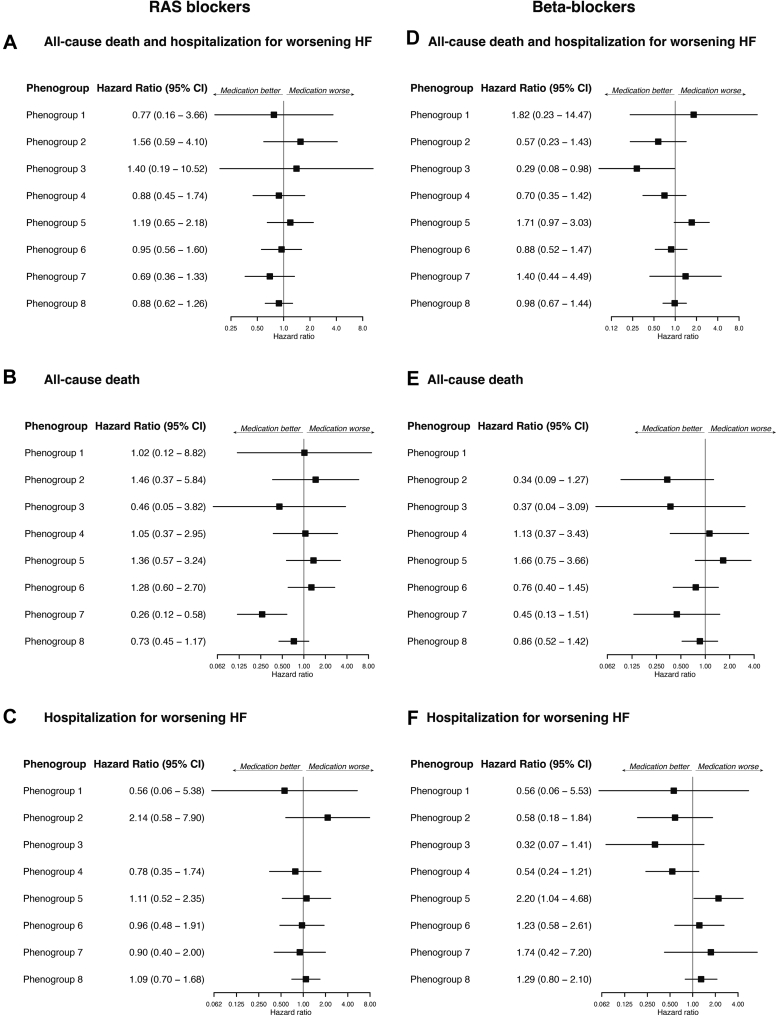
Clinical Outcomes by Phenogroups and Receiving RAS Blockers and Beta-Blockers (A) All-cause death and HF hospitalization. (B) All-cause death. (C) HF hospitalization. There were no events in patients without RAS blockers in phenogroup 3. Beta-blockers; (D) All-cause death and HF hospitalization. (E) All-cause death. There were no events in patients without beta-blockers in phenogroup 1. (F) HF hospitalization. RAS = renin-angiotensin system; other abbreviations as in Figure 2.

**Figure 5:**
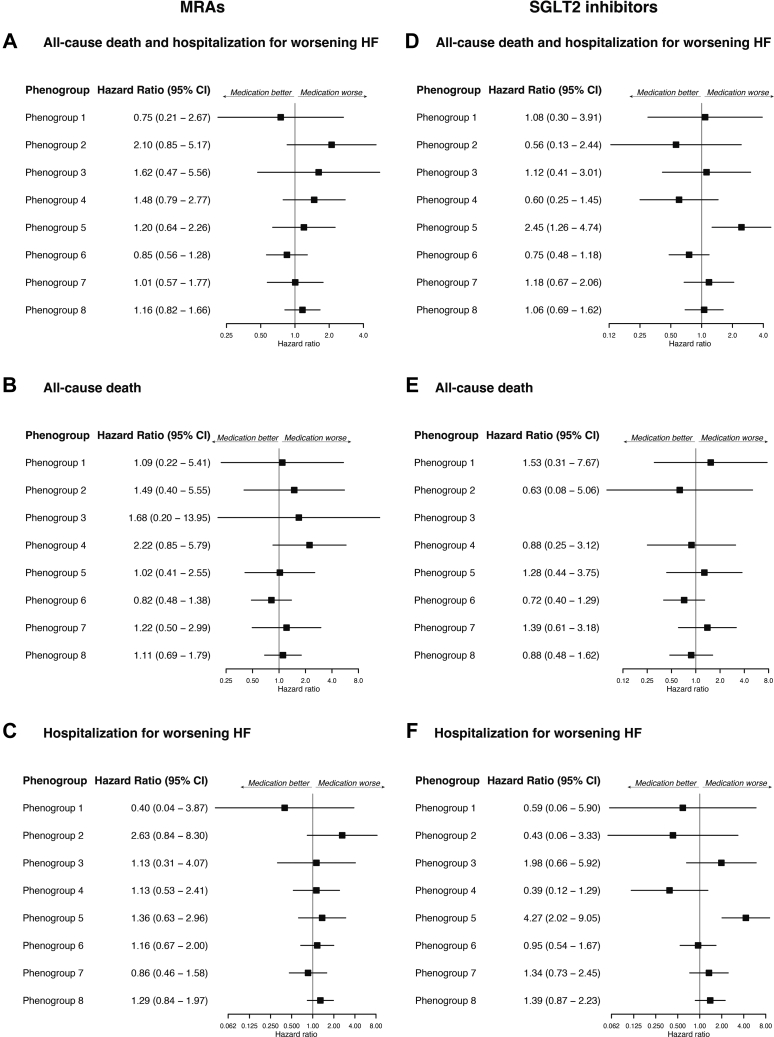
Clinical Outcomes by Phenogroups and Receiving MRAs and SGLT2 Inhibitors (A) All-cause death and HF hospitalization. (B) All-cause death. (C) HF hospitalization. SGLT2 inhibitors; (D) All-cause death and HF hospitalization. (E) All-cause death. There were no events in patients without SGLT2 inhibitors in phenogroup 3. (F) HF hospitalization. MRA = mineralocorticoid receptor antagonists; SGLT2 = sodium-glucose cotransporter 2; other abbreviations as in Figure 2.

Table 2 shows the baseline medications used across phenogroups. Overall, 1,640/2,220 (73.9%), 1,694/2,220 (76.3%), 1,096/2,230 (49.1%), and 713/2,229 (32.0%) patients received RAS blockers, beta-blockers, mineralocorticoid receptor antagonists, and SGLT2 inhibitors, respectively. Associations between phenogroups and primary and secondary outcomes, with or without each medication, are summarized in Figures 4 and 5. In phenogroup 7, patients receiving RAS blockers showed a significantly lower all-cause mortality. In phenogroup 5, patients receiving beta-blockers had a significantly higher rate of HF readmissions. Additionally, in phenogroup 5, patients receiving SGLT2 inhibitors had significantly higher rates of primary outcome and HF readmissions. Even after multivariable adjustment, patients receiving beta-blockers or SGLT2 inhibitors in phenogroup 5 had a significantly higher risk of HF hospitalization (HR: 2.71; 95% CI: 1.06-6.97 and HR: 5.40; 95% CI: 2.36-12.32, respectively) (Supplemental Table 4). Among patients with atrial fibrillation in phenogroup 5, the use of beta-blockers was not associated with significant differences in the incidence of HF hospitalization. In contrast, in those without atrial fibrillation, the use of beta-blockers had a significantly higher incidence of HF hospitalization (Supplemental Figure 17).

**Table 2:** Baseline Medications

	All Patients (N = 2,301)	Phenogroup 1	Phenogroup 2	Phenogroup 3	Phenogroup 4	Phenogroup 5	Phenogroup 6	Phenogroup 7	Phenogroup 8	*P* Value
		Younger Age With Obesity (n = 245)	Less Structural Abnormality and Comorbidity (n = 329)	Younger Age With LV Dilation (n = 251)	LV Hypertrophy (n = 336)	Older Age With Small LV and Diastolic Dysfunction (n = 256)	ICM (n = 361)	Advanced LV Remodeling and VAs (n = 177)	Atrial Myopathy (n = 346)	
RAS blockers	1,640 (73.9)	190 (86.8)	184 (58.6)	222 (90.2)	236 (72.4)	174 (69.0)	284 (80.7)	147 (85.0)	203 (60.1)	<0.001
ACE inhibitors	575 (26.2)	62 (28.2)	58 (18.8)	122 (50.4)	72 (22.4)	36 (14.3)	100 (28.9)	77 (44.8)	48 (14.2)	<0.001
ARBs	1,071 (48.8)	128 (59.8)	126 (40.8)	102 (42.7)	166 (51.2)	138 (55.0)	185 (53.2)	70 (41.4)	156 (46.0)	<0.001
ARNI	367 (16.5)	53 (24.1)	35 (11.1)	58 (23.6)	47 (14.3)	27 (10.6)	78 (22.2)	22 (12.9)	47 (13.9)	<0.001
Beta-blockers	1,694 (76.3)	182 (82.4)	218 (69.6)	232 (94.3)	257 (78.6)	128 (50.8)	281 (80.1)	160 (92.0)	236 (70.2)	<0.001
Carvedilol	660 (29.7)	93 (42.1)	70 (22.4)	104 (42.3)	75 (22.9)	39 (15.5)	135 (38.5)	87 (50.0)	57 (17.0)	<0.001
Bisoprolol	1,024 (46.1)	89 (40.3)	145 (46.3)	128 (52.0)	180 (55.0)	87 (34.5)	144 (41.0)	73 (42.0)	178 (53.0)	<0.001
MRAs	1,096 (49.1)	106 (48.0)	113 (35.9)	193 (78.5)	136 (41.5)	62 (24.4)	186 (52.8)	131 (75.3)	169 (49.7)	<0.001
Spironolactone	816 (36.6)	68 (30.8)	80 (25.4)	152 (61.8)	91 (27.7)	46 (18.1)	145 (41.2)	101 (58.0)	133 (39.1)	<0.001
Eplerenone	269 (12.1)	36 (16.3)	30 (9.5)	42 (17.1)	41 (12.5)	16 (6.3)	41 (11.6)	30 (17.3)	33 (9.7)	<0.001
SGLT2 inhibitors	713 (32.0)	99 (44.8)	62 (19.7)	100 (40.7)	102 (31.2)	46 (18.1)	153 (43.5)	54 (31.0)	97 (28.5)	<0.001

Values are n (%).

ACE = angiotensin converting enzyme; ARB = angiotensin II receptor blockers; ARNI = angiotensin receptor neprilysin inhibitor; MRA = mineralocorticoid receptor antagonist; RAS = renin-angiotensin system; SGLT2 = sodium-glucose cotransporter 2; other abbreviations as in Table 1.

**Central Illustration:**
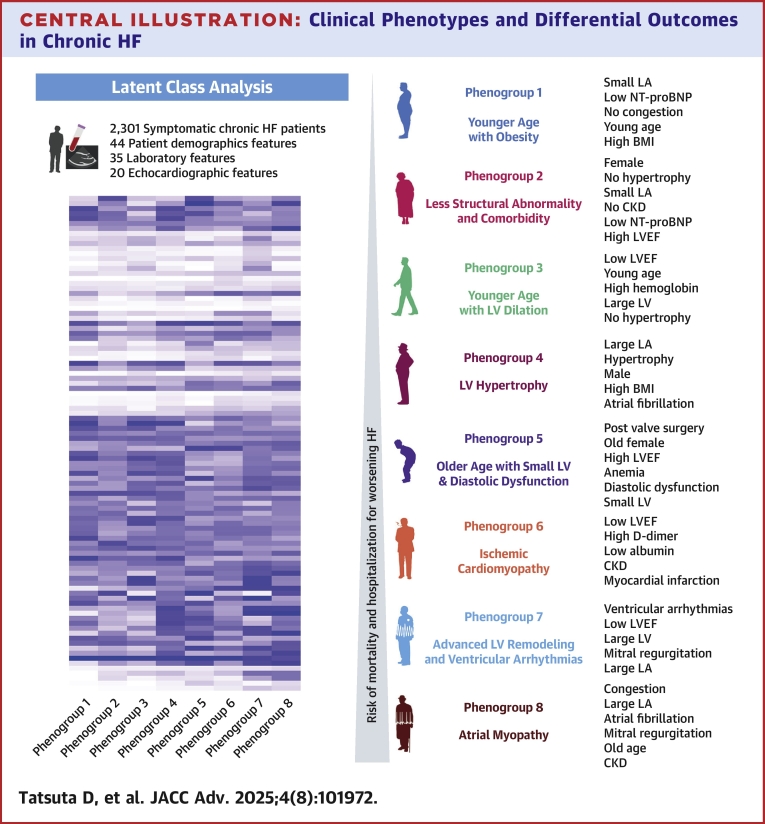

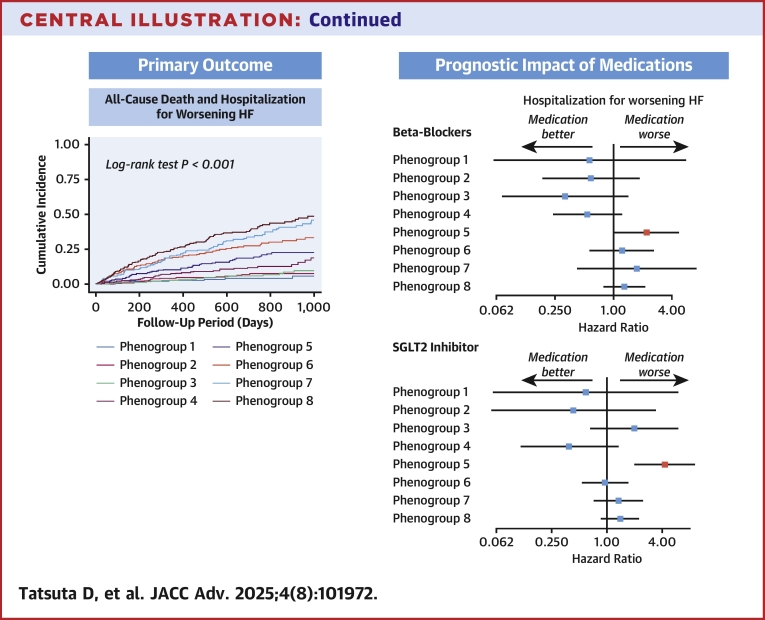
Clinical Phenotypes and Differential Outcomes in Chronic HF In this study, patients with chronic HF were classified into 8 phenogroups with distinct baseline characteristics and clinical outcomes using latent class analysis. Among the phenogroups, the phenogroup 5 characterized by older age with small LV and diastolic dysfunction demonstrated unfavorable outcomes with the use of beta-blockers or SGLT2 inhibitors. BMI = body mass index; CKD = chronic kidney disease; LVEF = left ventricular ejection fraction; other abbreviations as in Figures 2 and 5.

### Internal validation

The agreement between the phenogroup assignments in the internal validation and derivation cohorts was 0.602 (95% CI: 0472-0.751) (Supplemental Figure 18). The clinical characteristics of each phenogroup in the internal validation data sets were similar to those in the derivation cohort (Supplemental Figures 19 to 26). In addition, Kaplan-Meier analysis of the internal validation cohort showed a significant difference in each clinical outcome across the phenogroups (Supplemental Figure 27), consistent with the findings from the derivation cohort.

## Discussion

In this study, we performed LCA and successfully classified the studied patients into 8 phenogroups, using data from a Japanese multicenter registry conducted in the era of current clinical guidelines. We found that prognosis differed across these phenogroups identified through LCA. Notably, in patients classified into phenogroup 5 (characterized by very old age, small LV size, and diastolic dysfunction), the prescription of beta-blockers or SGLT2 inhibitors was associated with an increased risk of adverse events, particularly hospitalization due to worsening HF. These findings suggest that the LCA approach is useful for stratifying complex heterogeneity and the risk of adverse events and identifying potential subgroups showing specific responses to medical therapies in patients with chronic HF beyond the conventional classification based on LVEF (Central Illustration).

HF is a heterogeneous syndrome marked by dynamic, patient-specific changes in functional and structural biomarkers during disease progression. Therefore, phenotyping based solely on LVEF reveals a spectrum of overlapping HF phenotypes, highlighting the limitations of LVEF-based classification, as supported by epidemiological, clinical, and pathophysiological data.^8^ For instance, endothelial dysfunction, cardiomyocyte dysfunction, cardiomyocyte injury, systolic and diastolic LV dysfunction, LA dysfunction, myocardial fibrosis, and comorbidities such as renal dysfunction and pulmonary hypertension were observed regardless of the LVEF phenotype. In addition, there are bidirectional transitions in the LVEF owing to disease treatment and progression. Notably, 20% to 40% of patients with HFrEF transitioned to HF with mildly reduced ejection fraction (HFmrEF) or HFpEF over time, and 20% to 40% of patients with HFmrEF transitioned to HFpEF.^22^, ^23^, ^24^ In contrast, 10% to 40% of patients with HFpEF transitioned to HFmrEF or HFrEF, and 20% to 30% of patients with HFmrEF transitioned to HFrEF.^22^, ^23^, ^24^ Accordingly, LVEF-based HF phenotyping is clearly limited by accurate risk stratification and prediction of treatment effects in guideline-derived medical therapy.

Previous machine learning–based studies on HF phenotyping have primarily focused on patients with HFpEF or acute HF.^14^^,^^25^^,^^26^ Moreover, the number of clinical features used in these studies was relatively limited, with the largest one utilizing up to 63 variables.^26^ A recent study using a Japanese chronic HF cohort^16^ applied a random forest model to a broad spectrum of LVEF and used 56 clinical features to classify patients into 10 phenogroups. In contrast, in the present study, we analyzed a comprehensive Japanese chronic HF registry with a wide spectrum of patients with data on LVEF and 99 clinical features, including multiple biomarkers and echocardiographic parameters for phenotyping using LCA. This approach enabled the identification of previously undetectable phenotypes. First, we identified phenogroup 5, in which the use of beta-blockers and SGLT2 inhibitors was associated with a higher risk of HF hospitalization. Second, we characterized mixed LVEF clusters, such as phenogroup 1 (younger age with obesity), 3 (younger age with LV dilation), 4 (LV hypertrophy), and 6 (ischemic cardiomyopathy), highlighting LVEF-inconsistent but clinically meaningful phenogrouping. Additionally, none of the phenogroups were specifically associated with HFmrEF, highlighting the limited clinical utility of the HFmrEF category.

We also showed that clinical outcomes differed across the phenogroups identified through LCA, further extending the clinical utility of the machine learning approach using LCA. Although several clinical models for risk stratification in patients with HF have been proposed, these are limited by the linear assumptions between baseline characteristics and outcomes.^27^ However, our study demonstrated that LCA enabled nonlinear stratification of disease status by considering various pathophysiological backgrounds, allowing for the stratification of HF phenotypes with different outcomes. It is highly intriguing that phenogroups 5, 7, and 8—characterized particularly by poor prognosis—were identified in this study. Most patients in phenogroup 5 had HFpEF and a history of valvular surgery, suggesting underlying calcific aortic valve sclerosis. Inflammatory processes contribute to vascular calcification through activation of osteogenic signaling pathways.^28^ Patients with frailty often show elevated inflammatory markers.^29^ Consistently, phenogroup 5 showed relatively high C-reactive protein levels, suggesting that inflammation control may be important in this subgroup. Phenogroup 7 was characterized by advanced LV remodeling and ventricular arrhythmias. From a genetic perspective, mutations in genes such as *LMNA*, *SCN5A*, and *FLNC* have been reported as forms of dilated cardiomyopathy associated with electrical instability.^30^ However, in many cases, a clear genotype-phenotype correlation cannot be established.^31^ Although arrhythmias are typically caused by ion channel abnormalities, they also occur as part of the cardiomyopathy phenotype, due to gap junction remodeling and disrupted calcium homeostasis.^32^ Some of these alterations may reflect underlying epigenetic changes,^33^ suggesting a potential for earlier detection and risk stratification. We also identified a unique atrial myopathy phenotype (phenogroup 8), characterized by advanced atrial myopathy with specific features, including atrial fibrillation, LA dilation, preserved LVEF, and elevated pulmonary artery pressure, and the worst clinical outcomes among the phenogroups. LA myopathy is increasingly recognized as an important phenotypic trait of HFpEF.^34^^,^^35^ Reddy et al^36^ reported the impact of atrial fibrillation on HFpEF through invasive exercise testing and echocardiographic assessment. In their study, prolonged atrial fibrillation was associated with an increased LA volume, reduced LA compliance and reservoir strain, impaired right ventricular function, and elevated filling pressures. These changes were attributed to increased pericardial restraint caused by overall cardiac enlargement, ultimately resulting in decreased survival. Additionally, patients with phenogroup 8 had the largest right atrial diameter among all phenogroups. A previous study showed that patients with HFpEF and biatrial myopathy exhibited more pronounced abnormalities in biventricular structure and function, atrioventricular valve competence, pulmonary vascular disease, and cardiac reserve, which are associated with increased risks of mortality and HF hospitalization.^37^

Our study further highlighted that the effects of beta-blockers and SGLT2 inhibitors, as recommended by current guidelines, varied across the phenogroups in terms of clinical outcomes. In particular, their use was associated with a higher risk of hospitalization due to worsening HF in phenogroup 5 (characterized by very old age, small LV, and diastolic dysfunction). Moreover, this phenogroup was predominantly reclassified from HFpEF. In recent clinical trials targeting HFpEF, beta-blockers, which attenuate the chronotropic response, are used in approximately 80% of patients, despite the lack of clinical evidence supporting their efficacy.^38^ Reducing heart rate prolongs diastolic filling time, which increases ventricular volumes and pressures, elevating ventricular load.^39^ This subsequently increases myocardial wall stress, which may explain the elevated natriuretic peptide levels and worsened symptoms in HFpEF patients receiving beta-blockers.^40^^,^^41^ Regarding SGLT2 inhibitors, its use for patients with HFpEF is hypothesized to improve HF outcomes through mechanisms such as rapid reduction of pulmonary artery pressure, weight loss, and increased myocardial energy production.^42^ However, the average age in large clinical trials evaluating the efficacy of SGLT2 inhibitors for patients with HFpEF is approximately 70 years, which is significantly lower than the average age of 84 years observed in our patients in phenogroup 5.^12^^,^^43^ A previous study assessed the safety of SGLT2 inhibitors in older patients with type 2 diabetes and treatment discontinuation due to adverse events was found to be nearly twice as high in those aged ≥80 years compared with younger patients.^44^ Importantly, a lower baseline BMI and worsening renal function, features of phenogroup 5, were associated with an increased risk of SGLT2 inhibitor discontinuation.^44^ An important side effect of SGLT2 inhibitors is loss of body weight. A meta-analysis in patients with type 2 diabetes demonstrated that SGLT2 inhibitors not only reduced fat mass but also decreased muscle mass.^45^ Although SGLT2 inhibitors improve mitochondrial respiratory function,^46^ their effects on exercise tolerance in patients with HFpEF remain insufficiently established. In the EMPERIAL (Effect of EMPagliflozin on ExeRcise ability and HF symptoms In patients with chronic heArt faiLure) trial, SGLT2 inhibitors did not improve the 6-minute walking distance in patients with HF, including those with HFpEF.^47^ A recent study in a lean mouse model of hyperglycemia demonstrated that, although SGLT2 inhibitors led to an apparent improvement in exercise capacity due to fat loss in sedentary mice, true improvements in aerobic capacity were only observed in those undergoing exercise training, even after adjusting for lean body mass.^48^ These findings suggest the importance of combining SGLT2 inhibitors with exercise therapy, especially in lean or frail populations, such as phenogroup 5, while also indicating that the potential benefits of SGLT2 inhibitors in addressing diastolic dysfunction may be outweighed by the risk of adverse events, particularly in the context of the advanced age and frailty of this phenogroup.

### Study Limitations

The present study has some limitations that should be acknowledged. The data-driven LCA approach used for phenogrouping was highly influenced by cohort characteristics, and we were unable to perform external validation analyses for the classification system. Given the inherent nature of LCA, the number of phenogroups may vary across different analytic cohorts, potentially leading to different phenogroup patterns when analyzing other cohorts.^49^ Second, our cohort was comprised solely of Japanese patients, with a relatively high prevalence of HFpEF, thus necessitating confirmation of our findings in other populations. Third, several clinical parameters in this study had missing values, which may have introduced a selection bias. Although we used the missForest algorithm, which is a major imputation method for LCA classification, it remains limited in minimizing this bias. Finally, our analysis utilized only baseline data and did not account for transitional changes in the patients.

## Conclusions

In patients with chronic HF, we identified 8 phenogroups, each exhibiting unique clinical outcomes. This phenotypic classification introduces an innovative approach to risk stratification and holds the potential to enhance clinical decision-making processes.

Perspectives **COMPETENCY IN MEDICAL KNOWLEDGE:** The approach of using LCA to combine clinical features, multiple biomarkers, and detailed echocardiographic parameters identified 8 unique subgroups beyond the conventional classification based on LV ejection fraction in patients with chronic HF. Among these phenogroups, the phenogroup characterized by older age with small LV and diastolic dysfunction demonstrated unfavorable outcomes with the use of beta-blockers or SGLT2 inhibitors.

**TRANSLATIONAL OUTLOOK:** Understanding the molecular biological mechanisms underlying differential responses to medical therapies among the HF phenogroups could be enhanced by a phenotyping integrating clinical data and echocardiographic parameters with comprehensive multiomics analysis.

## Funding support and author disclosures

This study was supported by grants from the 10.13039/100007449Takeda Science Foundation (Dr Nagai), Mochida Memorial Foundation (Dr Nagai), 10.13039/501100006567Suhara Memorial Foundation (Dr Nagai), SENSIN Medical Research Foundation (Dr Nagai), 10.13039/100015639Japan Cardiovascular Research Foundation (Dr Nagai), 10.13039/501100008593Japan Foundation for Aging and Health (Dr Nagai), and 10.13039/100008732Uehara Memorial Foundation (Dr Nagai), and 10.13039/501100001691JSPS KAKENHI Grant-in-Aid for Scientific Research (B) (20H0367000) (Dr Anzai). Dr Nagai has received a research grant from 10.13039/501100012351Mitsubishi Tanabe Pharma Corp and honoraria from Kyowa Kirin Co, Ltd, Bayer Yakuhin, Ltd, Viatris Inc, and Boehringer Ingelheim Japan Co, Ltd. Dr Anzai has received a research grant from 10.13039/501100002973Daiichi Sankyo Co, Ltd; scholarship funds from Biotronik Japan Co, Ltd, Medtronic Japan Co, Ltd, Win International Co, Ltd, Medical System Network Co, Ltd, and Hokuyaku Takeyama Holdings, Inc; and honoraria from Daiichi Sankyo Co, Ltd, Ono Pharmaceutical Co, Ltd, Boehringer Ingelheim Japan Co, Ltd, Bayer’s Pharmaceuticals Co, Ltd, and Bristol Myers Squibb Co, Ltd. All other authors have reported that they have no relationships relevant to the contents of this paper to disclose.
