# Supplementary material for: Clinical Phenotyping and Treatment Response in Patients With Chronic Heart Failure
Source: JACC Adv. 2025 Jul 17;4(8):101972. doi: 10.1016/j.jacadv.2025.101972 (PMC12284680; doi:10.1016/j.jacadv.2025.101972)
Supplement: Supplementary data [file mmc1.pdf]

Supplemental Material

Clinical Phenotyping and Treatment Response in Patients with Chronic Heart Failure

Supplemental Material

Supplementary Table S1. Missing rate of all features.

Supplementary Table S2. Model fit evaluation information.

Supplementary Table S3. Cox proportional hazard models

Supplementary Table S4. Cox proportional hazards models for hospitalization for worsening HF in phenogroup 5

Supplementary Figure S1. Correlation between all variables used in latent class analysis.

Supplementary Figure S2. Bayesian information criterion analysis for the identification of the optimal number of phenogroups.

Supplementary Figure S3. Bayesian information criterion analysis for the identification of the optimal number of phenogroups in the first 10 of 1000 bootstrap iterations.

Supplementary Figure S4. Feature importance in Phenogroup 1

Supplementary Figure S5. Feature importance in Phenogroup 2

Supplementary Figure S6. Feature importance in Phenogroup 3

Supplementary Figure S7. Feature importance in Phenogroup 4

Supplementary Figure S8. Feature importance in Phenogroup 5

Supplementary Figure S9. Feature importance in Phenogroup 6

Supplementary Figure S10. Feature importance in Phenogroup 7

Supplementary Figure S11. Feature importance in Phenogroup 8

Supplementary Figure S12. Sankey diagram showing the association between left ventricular ejection fraction-based classification and eight phenogroups.

Supplementary Figure S13 Log-minus-log survival plots for proportional hazards assumption across phenogroups.

Supplementary Figure S14 Cumulative incidence of all-cause death and hospitalization for worsening heart failure.

Supplementary Figure S15 Cumulative incidence of all-cause death.

Supplementary Figure S16 Cumulative incidence of hospitalization for worsening heart failure.

Supplementary Figure S17. Cumulative incidence of worsening HF in Phenogroup 5 patients stratified by atrial fibrillation status.

Supplementary Figure S18. Total clustering heatmap of phenogroup concordance between derivation and bootstrap replications

Supplementary Figure S19. Feature importance in Phenogroup 1 based on bootstrap analysis

Supplementary Figure S20. Feature importance in Phenogroup 2 based on bootstrap analysis

Supplementary Figure S21. Feature importance in Phenogroup 3 based on bootstrap analysis

Supplementary Figure S22. Feature importance in Phenogroup 4 based on bootstrap analysis

Supplementary Figure S23 Feature importance in Phenogroup 5 based on bootstrap analysis

Supplementary Figure S24. Feature importance in Phenogroup 6 based on bootstrap analysis

Supplementary Figure S25. Feature importance in Phenogroup 7 based on bootstrap analysis

Supplementary Figure S26. Feature importance in Phenogroup 8 based on bootstrap analysis

Supplementary Figure S27. Cumulative incidence of clinical outcomes among the eight phenogroups identified by bootstrap analysis.

Supplementary Appendix S1. Participating centers and their principal investigators.

Supplementary Table S1. Missing rate of all features

Features	Missing count, n	Missing rate
Sex	0	0%
Atrial fibrillation	13	0.56%
Atrial flutter	14	0.61%
Ventricular arrhythmias	22	0.96%
HF admission within 1 year	27	1.17%
Pacemaker implantation	13	0.56%
Catheter ablation for AF	16	0.70%
Catheter ablation for non-AF	14	0.61%
CRT implantation	15	0.65%
ICD implantation	14	0.61%
Cerebrovascular Accident	17	0.74%
Hemodialysis	20	0.87%
Chronic obstructive pulmonary disease	15	0.65%
Bronchial asthma	17	0.74%
Chronic kidney disease	15	0.65%
Sleep apnea syndrome	16	0.70%
Depression	15	0.65%
Liver cirrhosis	19	0.83%
Myocardial infarction	14	0.61%
Coronary artery bypass grafting	16	0.70%
Percutaneous coronary intervention	18	0.78%
Hypertension	21	0.91%
Hyperuricemia	20	0.87%
Dyslipidemia	17	0.74%
Diabetes mellitus	28	1.22%
Valve surgery	21	0.91%
Malignancy	21	0.91%
Peripheral artery disease	20	0.87%
Dementia	20	0.87%
Smoke	40	1.74%
Alcohol	42	1.83%
Advance care planning	46	2%
Living alone	41	1.78%
Clinical frailty scale	77	3.35%
Hepatomegaly	28	1.22%
Orthopnea	28	1.22%
Rales >1/3 up lung fields	36	1.56%
Jugular vein distention	33	1.43%
Edema	35	1.52%
NYHA class III or IV	27	1.17%
IVC respiratory change	134	5.82%

Aortic regurgitation	100	4.35%
Mitral regurgitation	58	2.52%
Tricuspid regurgitation	56	2.43%
Aortic stenosis	175	7.61%
Mitral stenosis	129	5.61%
Age	0	0%
Body mass index	16	0.70%
Systolic blood pressure	15	0.65%
Diastolic blood pressure	15	0.65%
Heart rate	14	0.61%
EHFScBS	138	6%
White blood cell	19	0.83%
Red blood cell	19	0.83%
Hemoglobin	20	0.87%
Hematocrit	19	0.83%
Platelet	20	0.87%
Reticulocytes	150	6.52%
Lymphocyte	41	1.78%
Monocyte	44	1.91%
D-dimer	179	7.78%
Total protein	19	0.83%
Albumin	23	1%
Total bilirubin	21	0.91%
Aspartate aminotransferase	20	0.87%
Alanine aminotransferase	19	0.83%
Lactate dehydrogenase	21	0.91%
Gamma-glutamyl transpeptidase	20	0.87%
Alkaline phosphatase	29	1.26%
Estimated glomerular filtration rate	19	0.83%
Uric acid	20	0.87%
Sodium	19	0.83%
Potassium	19	0.83%
Chloride	19	0.83%
Transferrin saturation	132	5.74%
Ferritin	120	5.22%
Blood sugar	26	1.13%
Hemoglobin A1c	46	2%
High-sensitivity troponin I	1976	85.88%
High-sensitivity troponin T	821	35.68%
Total cholesterol	98	4.26%
Triglyceride	56	2.43%
High-density lipoprotein	47	2.04%
Low-density lipoprotein	53	2.30%

C-reactive protein	49	2.13%
B-type Natriuretic Peptide	1628	70.75%
Free Triiodothyronine	267	11.60%
Free Thyroxine	141	6.13%
Thyroid Stimulating Hormone	143	6.21%
Erythropoietin	354	15.38%
Plasma renin activity	358	15.56%
Aldosterone	364	15.82%
N-terminal pro B-type natriuretic peptide	165	7.17%
LV end-diastolic diameter	56	2.43%
LV end-systolic diameter	76	3.30%
LV interventricular septal thickness	58	2.52%
LV posterior wall thickness	173	7.52%
LA diameter	58	2.52%
LV end-diastolic volume	376	16.34%
LV end-systolic volume	284	12.34%
LV ejection fraction	65	2.82%
LA volume	272	11.82%
LA volume index	326	14.17%
RV end-diastolic diameter at the base	1024	44.50%
RV end-diastolic diameter at the mid	1020	44.33%
Tricuspid valve diameter	1468	63.80%
Tricuspid annular plane systolic excursion	953	41.42%
RA diameter	705	30.64%
IVC diameter	170	7.39%
E-wave	269	11.69%
A-wave	801	34.81%
A-wave duration	1442	62.67%
E-wave deceleration time	301	13.08%
s’septal	1207	52.46%
e’septal	398	17.30%
a’septal	1350	58.67%
s’lateral	1219	52.98%
e’lateral	631	27.42%
a’lateral	1436	62.41%
RVs'	1437	62.45%
E/e’average	360	15.65%
Tricuspid regurgitation pressure gradient	442	19.21%
Pulmonary venous systole	1438	62.49%
Pulmonary venous diastole	1384	60.15%
Pulmonary venous atrial	1565	68.01%
Pulmonary venous atrial diastole	1612	70.06%
Pulmonary regurgitation early diastolic pressure gradient	1491	64.80%

Pulmonary regurgitation end diastolic pressure gradient	1380	59.97%
Stroke volume	338	14.69%
LV mass	180	7.82%
LV mass index	192	8.34%
Relative wall thickness	178	7.74%
Systolic pulmonary artery pressure	571	24.82%

AF, atrial fibrillation; CRT, cardiac resynchronization therapy; EHFScBS, European Heart Failure Self-care Behavior Scale; HF, heart failure; ICD, implantable cardioverter-defibrillator; IVC, inferior vena cava; LA, left atrium; LV, left ventricle; NYHA, New York Heart Association; RA, right atrium; RV, right ventricle.

Supplementary Table S2. Model fit evaluation information.

	Log-Likelihood	AIC	BIC	Likelihood Ratio Statistic	Classification Error	Entropy
2-Phenogroups	-120984.7141	242367.4282	243509.907	206344.8902	0.044446378	0.847053913
3-Phenogroups	-119572.4597	239742.9194	241459.508	203520.3814	0.067543059	0.848561879
4-Phenogroups	-118617.0607	238032.1215	240322.82	201609.5835	0.07919261	0.857820841
5-Phenogroups	-117935.958	236869.9159	239734.7244	200247.3779	0.087007375	0.86117431
6-Phenogroups	-117181.2386	235560.4771	238999.3955	198737.9391	0.090575665	0.870235231
7-Phenogroups	-116621.7192	234641.4384	238654.4667	197618.9004	0.09440108	0.874333935
8-Phenogroups	-116170.356	233938.7119	238525.8501	196716.1739	0.094280866	0.880688771
9-Phenogroups	-116156.2684	234110.5368	239271.7849	196687.9988	0.098844495	0.883196405
10-Phenogroups	-115667.3533	233332.7065	239068.0645	195710.1685	0.092472121	0.891892548
11-Phenogroups	-115035.906	232269.8121	238579.28	194447.274	0.089841226	0.896786847
12-Phenogroups	-115056.26	232510.52	239394.0978	194487.982	0.08960364	0.901376269
13-Phenogroups	-114476.4061	231550.8123	239008.5	193328.2743	0.093835181	0.899489494
14-Phenogroups	-114388.6917	231575.3834	239607.181	193152.8454	0.088569269	0.904337514
15-Phenogroups	-114296.1541	231590.3081	240196.2157	192967.7701	0.085569789	0.910092766

AIC, Akaike Information Criterion; BIC, Bayesian Information Criterion

Supplementary Table S3. Cox proportional hazard models

	All-cause death and hospitalization for worsening HF		All-cause death		Hospitalization for worsening HF	
Variable	HR (95% CI)	*P value*	HR (95% CI)	*P value*	HR (95% CI)	*P value*
Phenogroup 1	1.00 (reference)	-	1.00 (reference)	-	1.00 (reference)	-
Phenogroup 2	1.32 (0.61-2.84)	0.475	1.02 (0.36-2.86)	0.975	2.10 (0.68-6.52)	0.198
Phenogroup 3	1.49 (0.69-3.21)	0.308	0.84 (0.28-2.50)	0.754	2.84 (0.93-8.63)	0.066
Phenogroup 4	2.92 (1.46-5.85)	0.002	2.16 (0.86-5.45)	0.101	5.08 (1.78-14.51)	0.002
Phenogroup 5	4.40 (2.23-8.66)	<0.001	3.41 (1.40-8.28)	0.007	7.09 (2.51-20.04)	<0.001
Phenogroup 6	7.31 (3.80-14.04)	<0.001	7.07 (3.04-16.4)	<0.001	10.87 (3.93-30.05)	<0.001
Phenogroup 7	9.07 (4.67-17.63)	<0.001	5.49 (2.29-13.17)	<0.001	18.47 (6.69-51.02)	<0.001
Phenogroup 8	10.61 (5.57-20.22)	<0.001	8.61 (3.74-19.83)	<0.001	18.35 (6.73-50.04)	<0.001

CI, confidence interval; HF, heart failure; HR, hazard ratio

Supplementary Table S4. Cox proportional hazards models for heart failure hospitalization in phenogroup 5

Model	Beta-blockers<br>HR (95% CI)	SGLT2 inhibitors<br>HR (95% CI)
Crude	2.20 (1.04–4.68)	4.27 (2.02–9.05)
Model 1: MAGGIC-HF risk model	2.57 (1.17–5.66)	4.63 (2.15–9.98)
Model 2: MAGGIC-HR risk model + NT-pro BNP	2.71 (1.06–6.97)	5.40 (2.36–12.32)

CI, confidence interval; HF, heart failure; HR, hazard ratio; NT-proBNP, N-terminal pro-brain natriuretic peptide; MAGGIC-HR, Meta-Analysis Global Group in Chronic Heart Failure

Supplementary Figure S1 Correlation between all variables used in latent class analysis

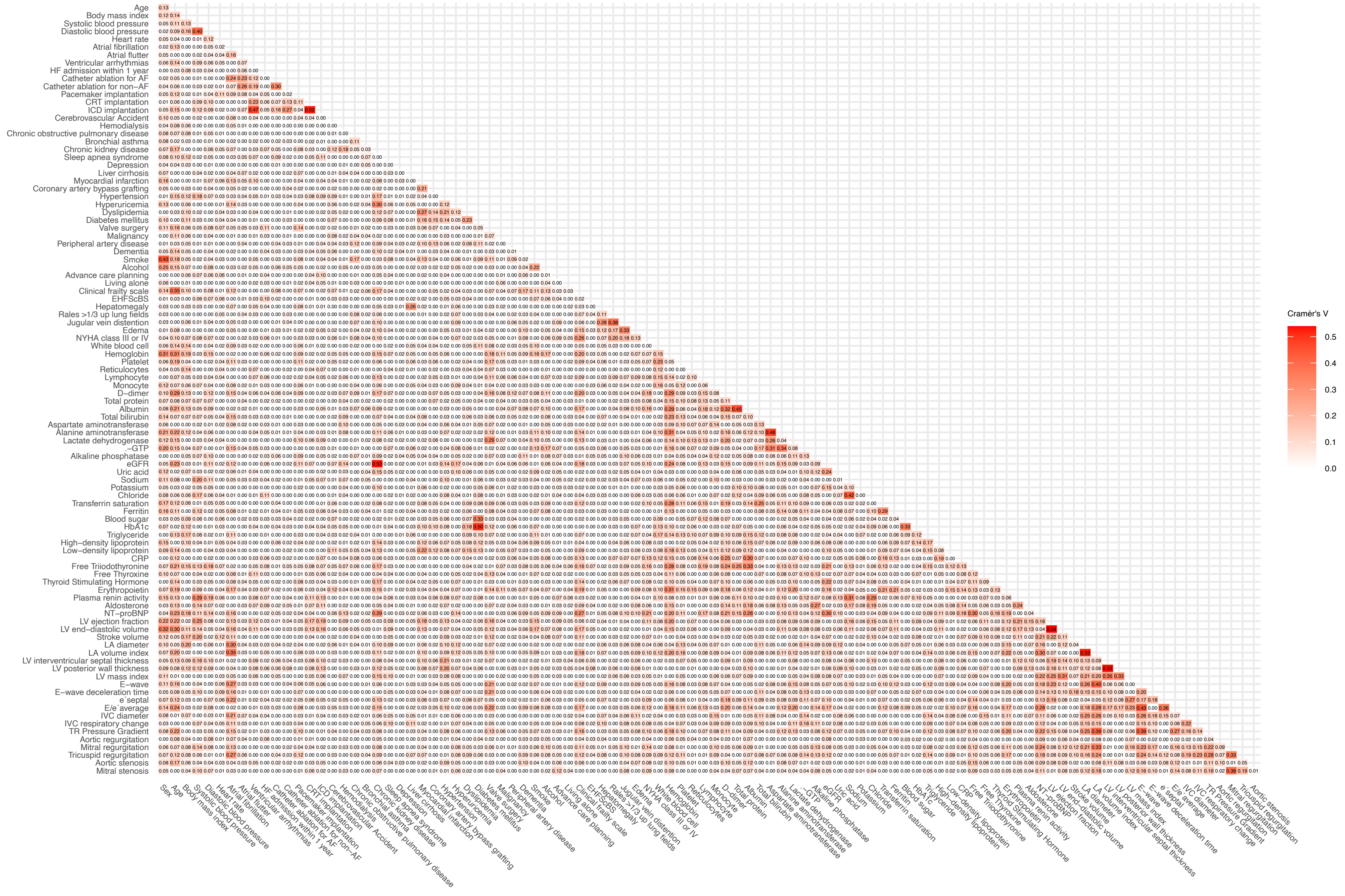

AF, atrial fibrillation; BMI, body mass index; CRT, cardiac resynchronization therapy; CRP, C-reactive protein; EHFScBS, European Heart Failure Self-care Behaviour Scale; eGFR, estimated glomerular filtration rate; γ-GTP, gamma-glutamyl transpeptidase; HbA1c, hemoglobin A1c; HF, heart failure; ICD, implantable cardioverter-defibrillator; IVC, inferior vena cava; LA, left atrium; LV, left ventricle; NT-proBNP, N-terminal pro B-type natriuretic peptide; NYHA, New York Heart Association; TR, tricuspid regurgitation.

Supplementary Figure S2 Bayesian information criterion analysis for the identification of the optimal number of phenogroups.

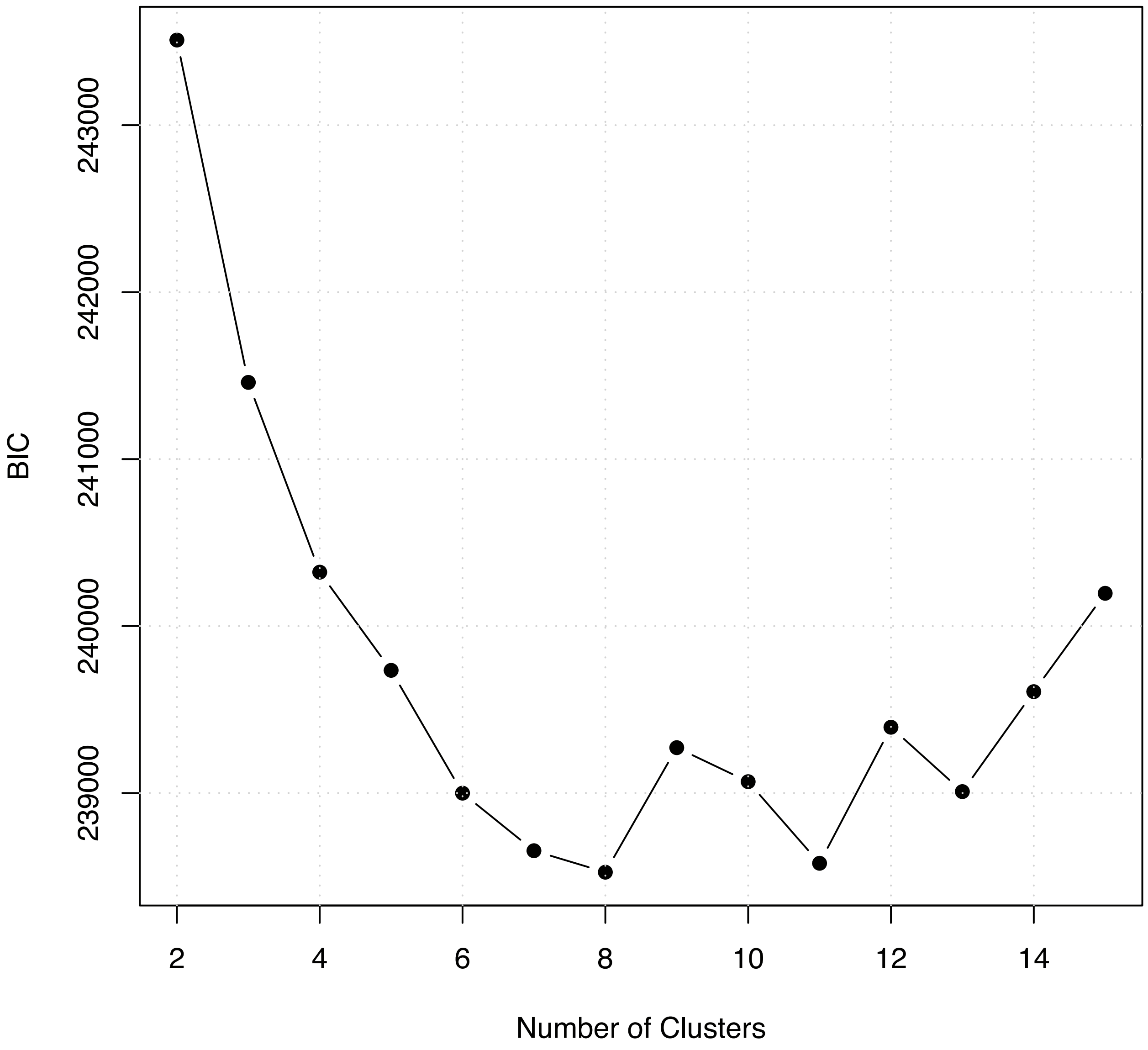

BIC, Bayesian information criterion

Supplementary Figure S3. Bayesian information criterion analysis for the identification of the optimal number of phenogroups in the first 10 of 1000 bootstrap iterations.

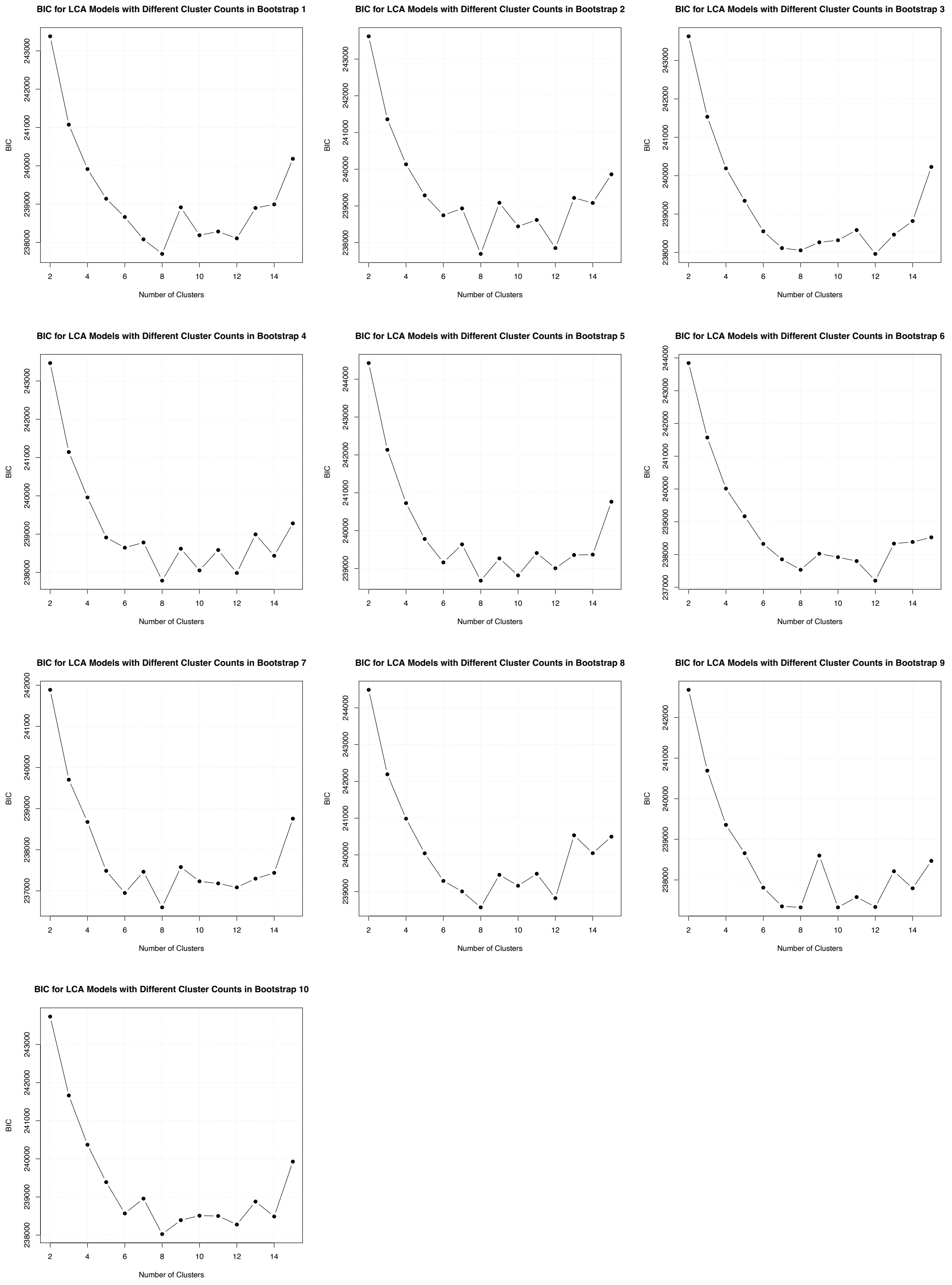

BIC, Bayesian information criterion

Supplementary Figure S4. Feature importance in Phenogroup 1

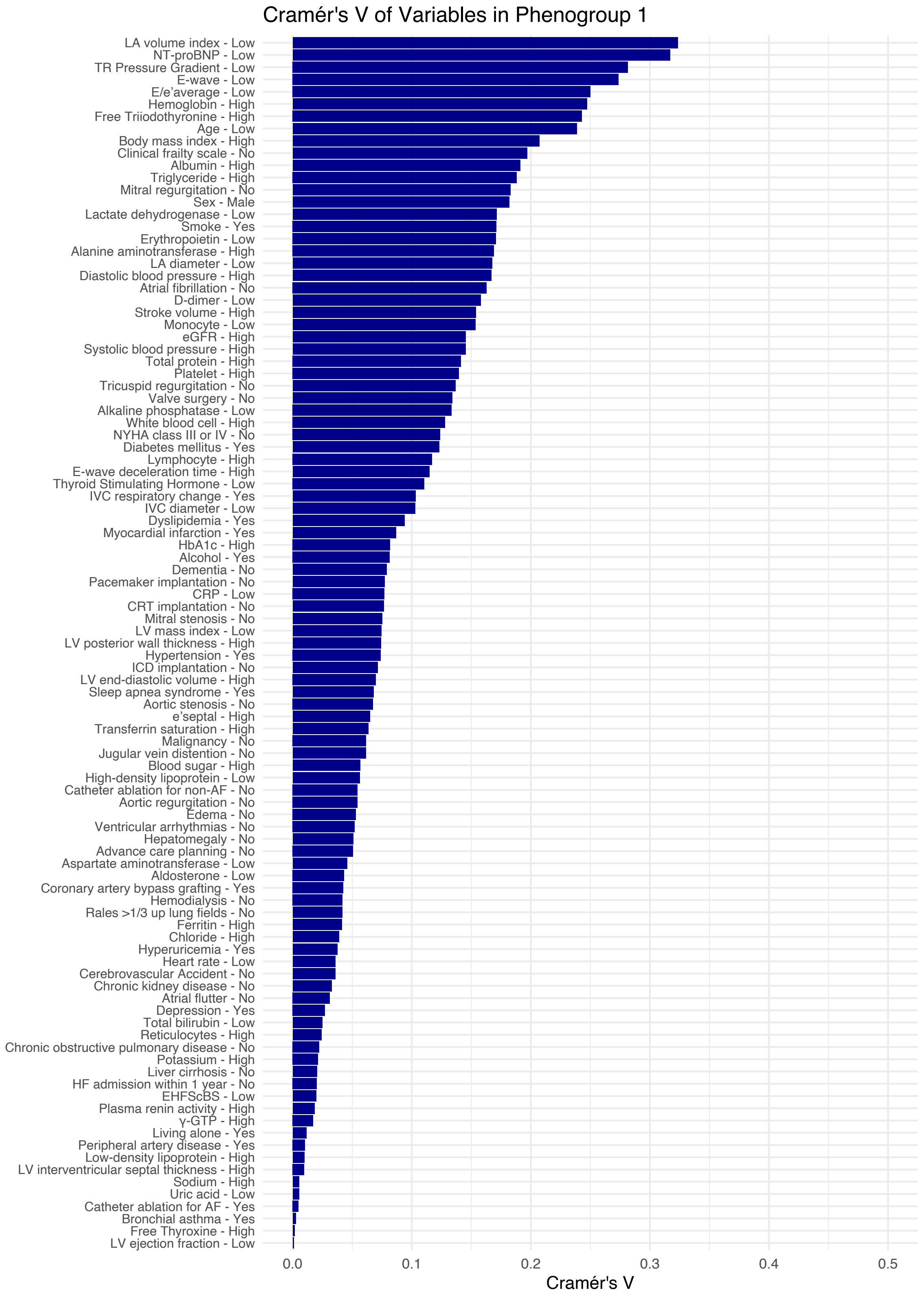

AF, atrial fibrillation; CRT, cardiac resynchronization therapy; CRP, C-reactive protein; EHFScBS, European Heart Failure Self-care Behaviour Scale; eGFR, estimated glomerular filtration rate; γ-GTP, gamma-glutamyl transpeptidase; HbA1c, hemoglobin A1c; HF, heart failure; ICD, implantable cardioverter-defibrillator; IVC, inferior vena cava; LA, left atrium; LV, left ventricle; NT-proBNP, N-terminal pro B-type natriuretic peptide; NYHA, New York Heart Association; TR, tricuspid regurgitation.

Supplementary Figure S5. Feature importance in Phenogroup 2

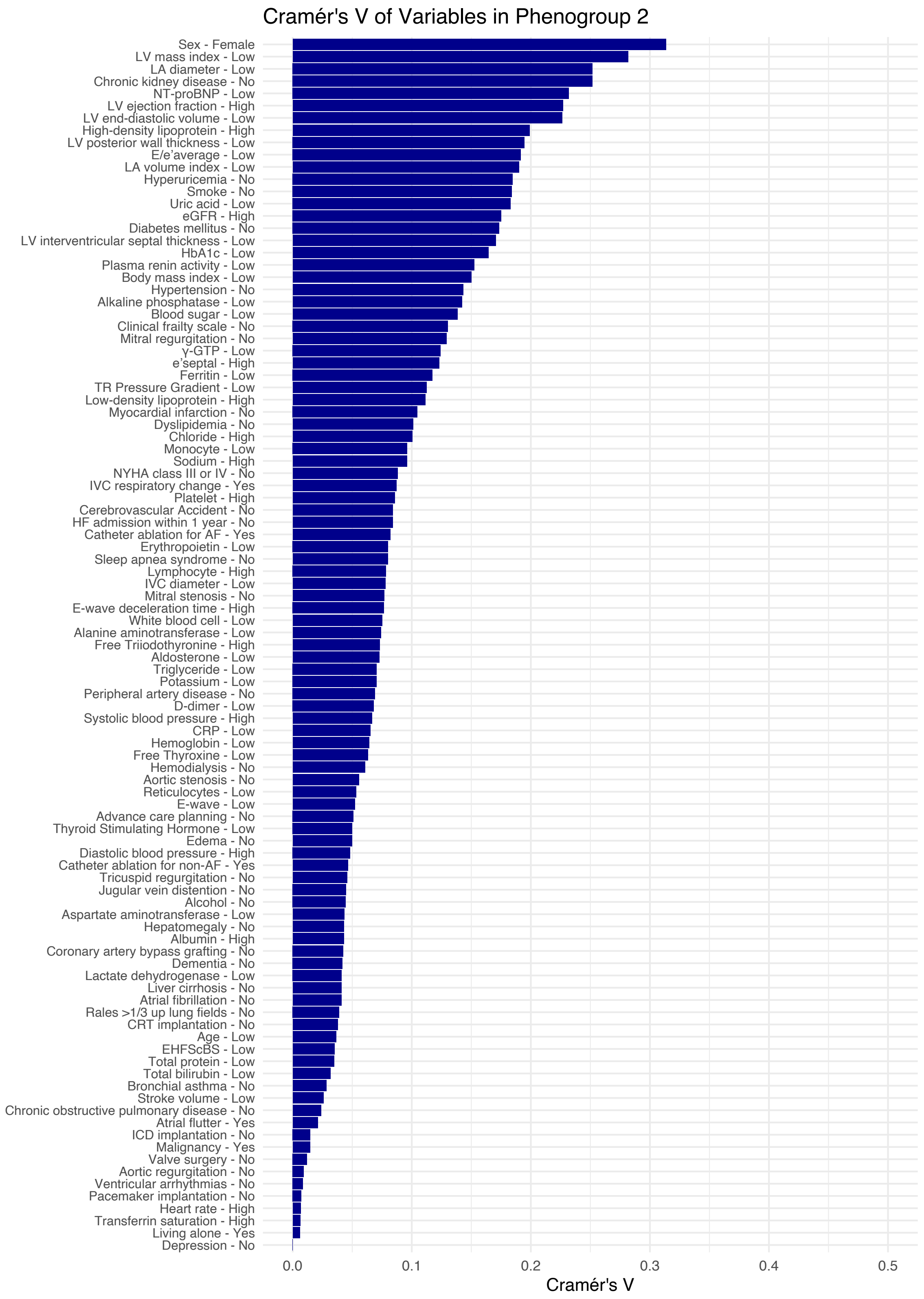

AF, atrial fibrillation; CRT, cardiac resynchronization therapy; CRP, C-reactive protein; EHFScBS, European Heart Failure Self-care Behaviour Scale; eGFR, estimated glomerular filtration rate; γ-GTP, gamma-glutamyl transpeptidase; HbA1c, hemoglobin A1c; HF, heart failure; ICD, implantable cardioverter-defibrillator; IVC, inferior vena cava; LA, left atrium; LV, left ventricle; NT-proBNP, N-terminal pro B-type natriuretic peptide; NYHA, New York Heart Association; TR, tricuspid regurgitation.

Supplementary Figure S6. Feature importance in Phenogroup 3

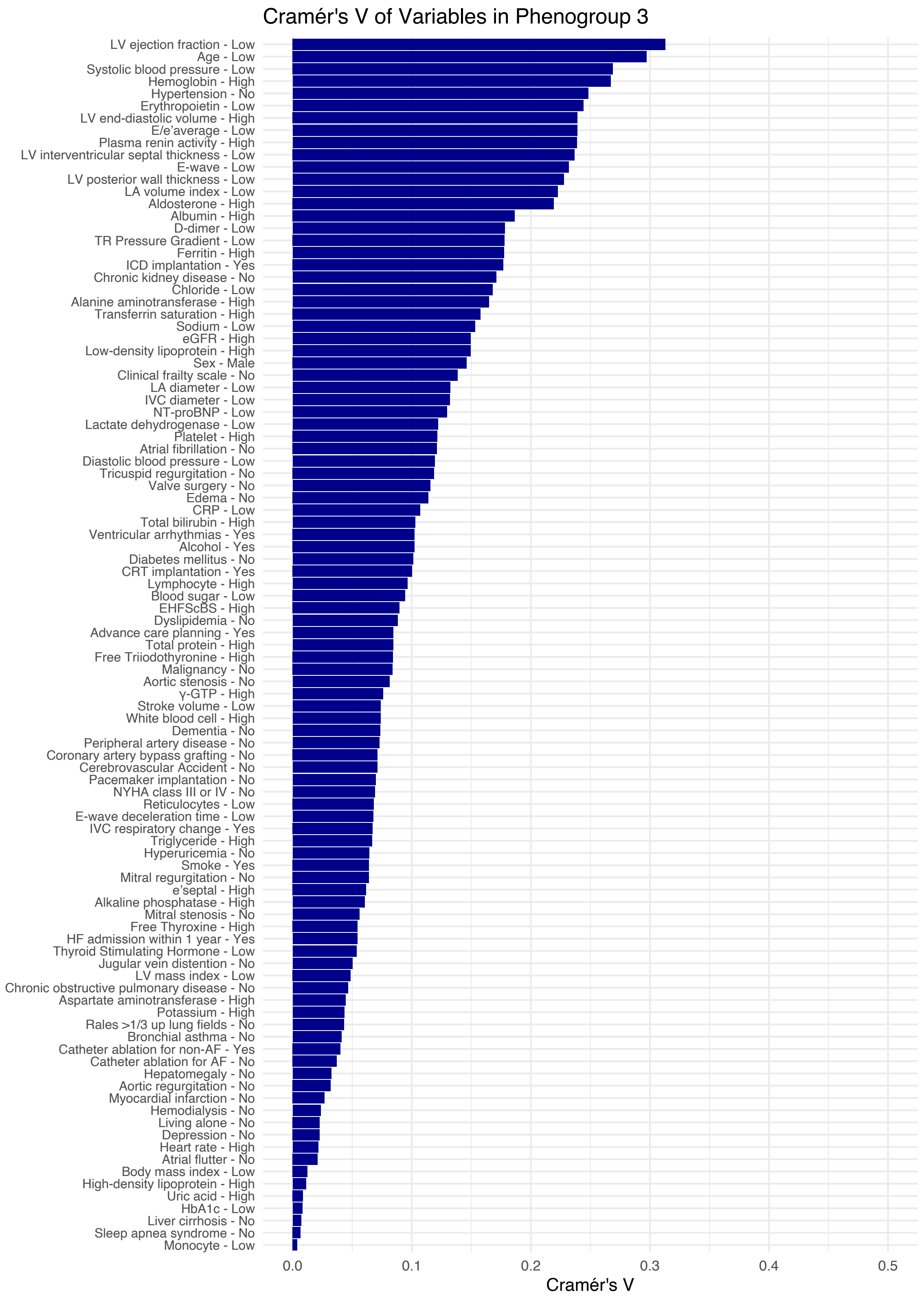

AF, atrial fibrillation; CRT, cardiac resynchronization therapy; CRP, C-reactive protein; EHFScBS, European Heart Failure Self-care Behaviour Scale; eGFR, estimated glomerular filtration rate; γ-GTP, gamma-glutamyl transpeptidase; HbA1c, hemoglobin A1c; HF, heart failure; ICD, implantable cardioverter-defibrillator; IVC, inferior vena cava; LA, left atrium; LV, left ventricle; NT-proBNP, N-terminal pro B-type natriuretic peptide; NYHA, New York Heart Association; TR, tricuspid regurgitation.

Supplementary Figure S7. Feature importance in Phenogroup 4

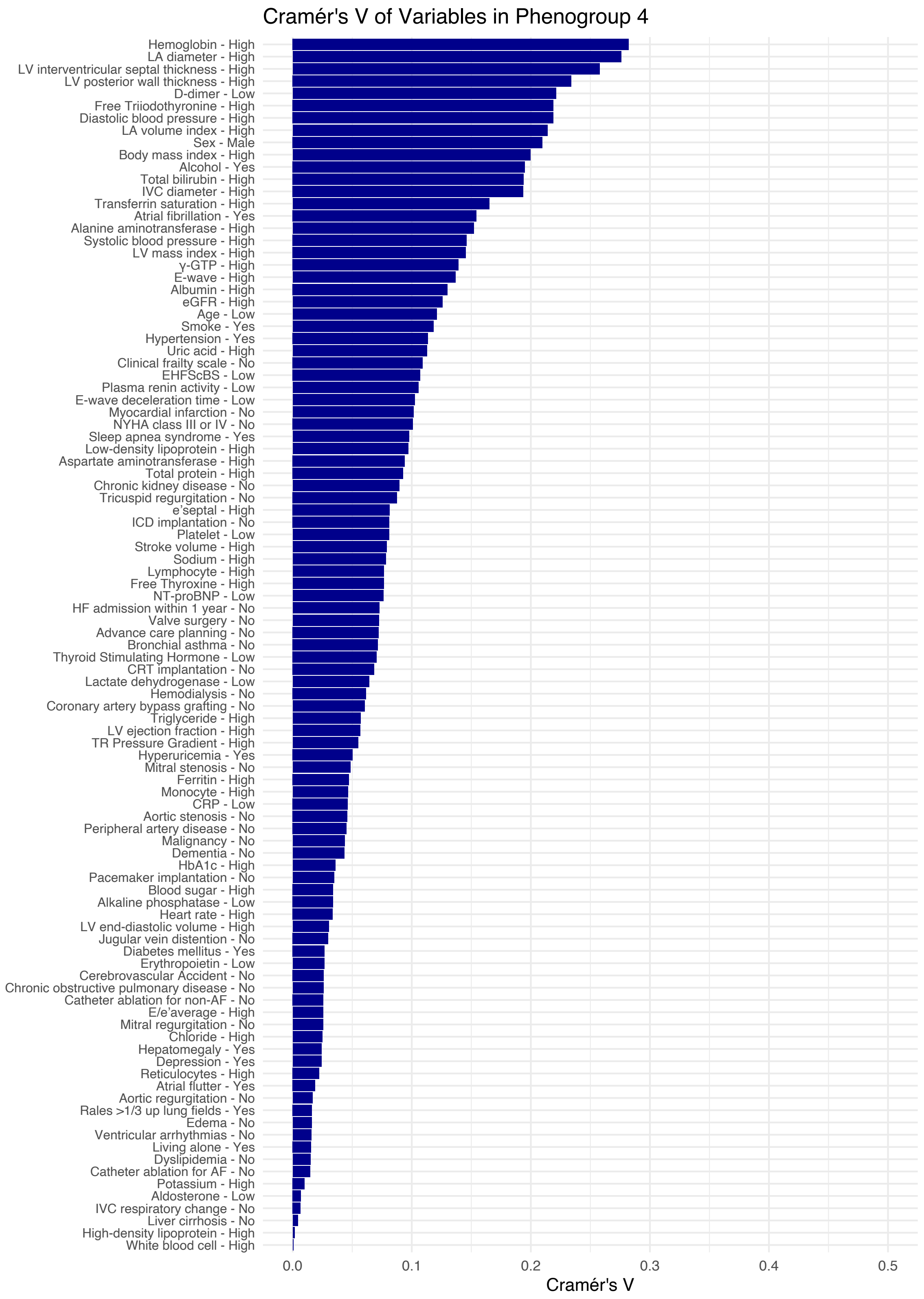

AF, atrial fibrillation; CRT, cardiac resynchronization therapy; CRP, C-reactive protein; EHFScBS, European Heart Failure Self-care Behaviour Scale; eGFR, estimated glomerular filtration rate; γ-GTP, gamma-glutamyl transpeptidase; HbA1c, hemoglobin A1c; HF, heart failure; ICD, implantable cardioverter-defibrillator; IVC, inferior vena cava; LA, left atrium; LV, left ventricle; NT-proBNP, N-terminal pro B-type natriuretic peptide; NYHA, New York Heart Association; TR, tricuspid regurgitation.

Supplementary Figure S8. Feature importance in Phenogroup 5

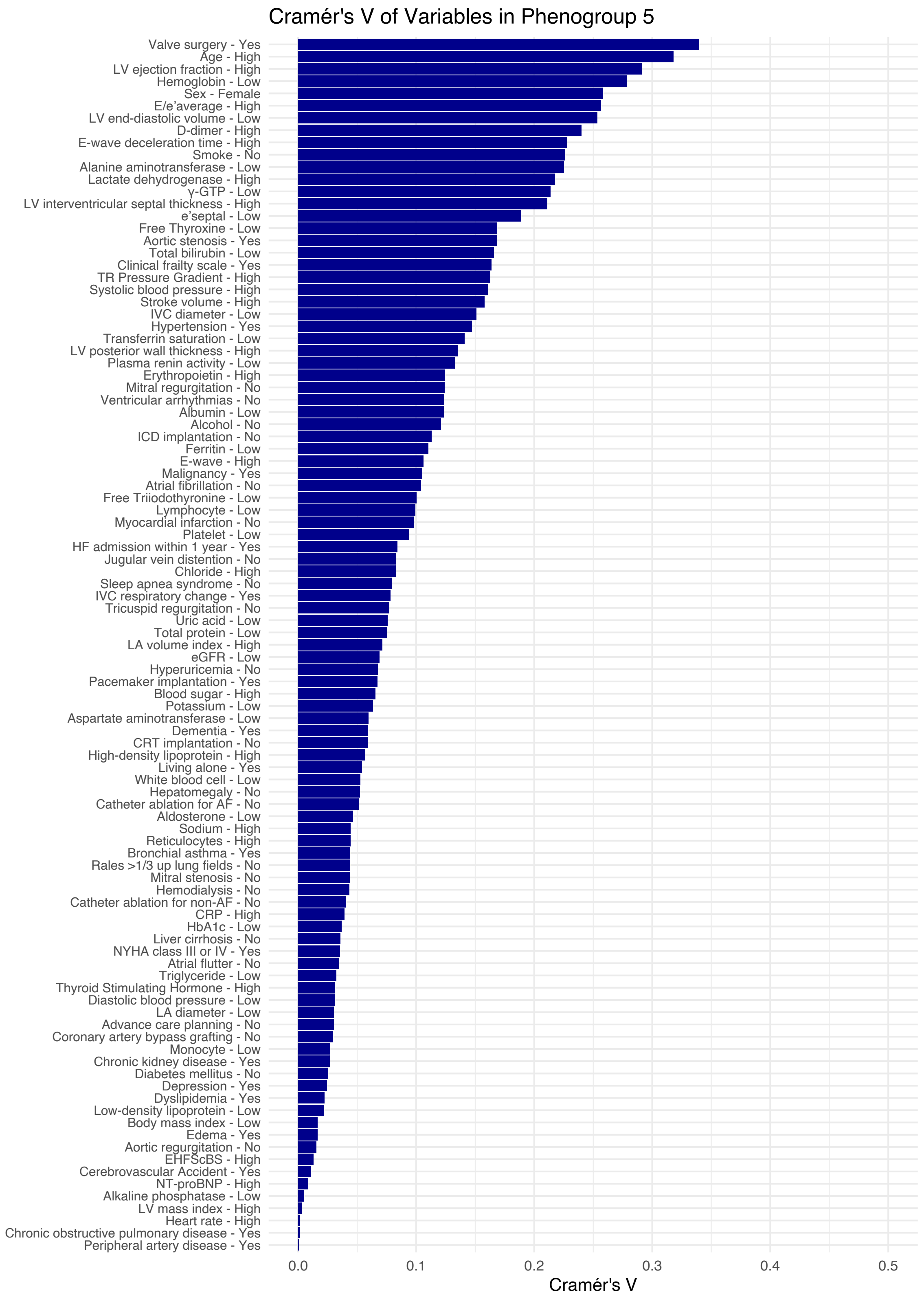

AF, atrial fibrillation; CRT, cardiac resynchronization therapy; CRP, C-reactive protein; EHFScBS, European Heart Failure Self-care Behaviour Scale; eGFR, estimated glomerular filtration rate; γ-GTP, gamma-glutamyl transpeptidase; HbA1c, hemoglobin A1c; HF, heart failure; ICD, implantable cardioverter-defibrillator; IVC, inferior vena cava; LA, left atrium; LV, left ventricle; NT-proBNP, N-terminal pro B-type natriuretic peptide; NYHA, New York Heart Association; TR, tricuspid regurgitation.

Supplementary Figure S9. Feature importance in Phenogroup 6

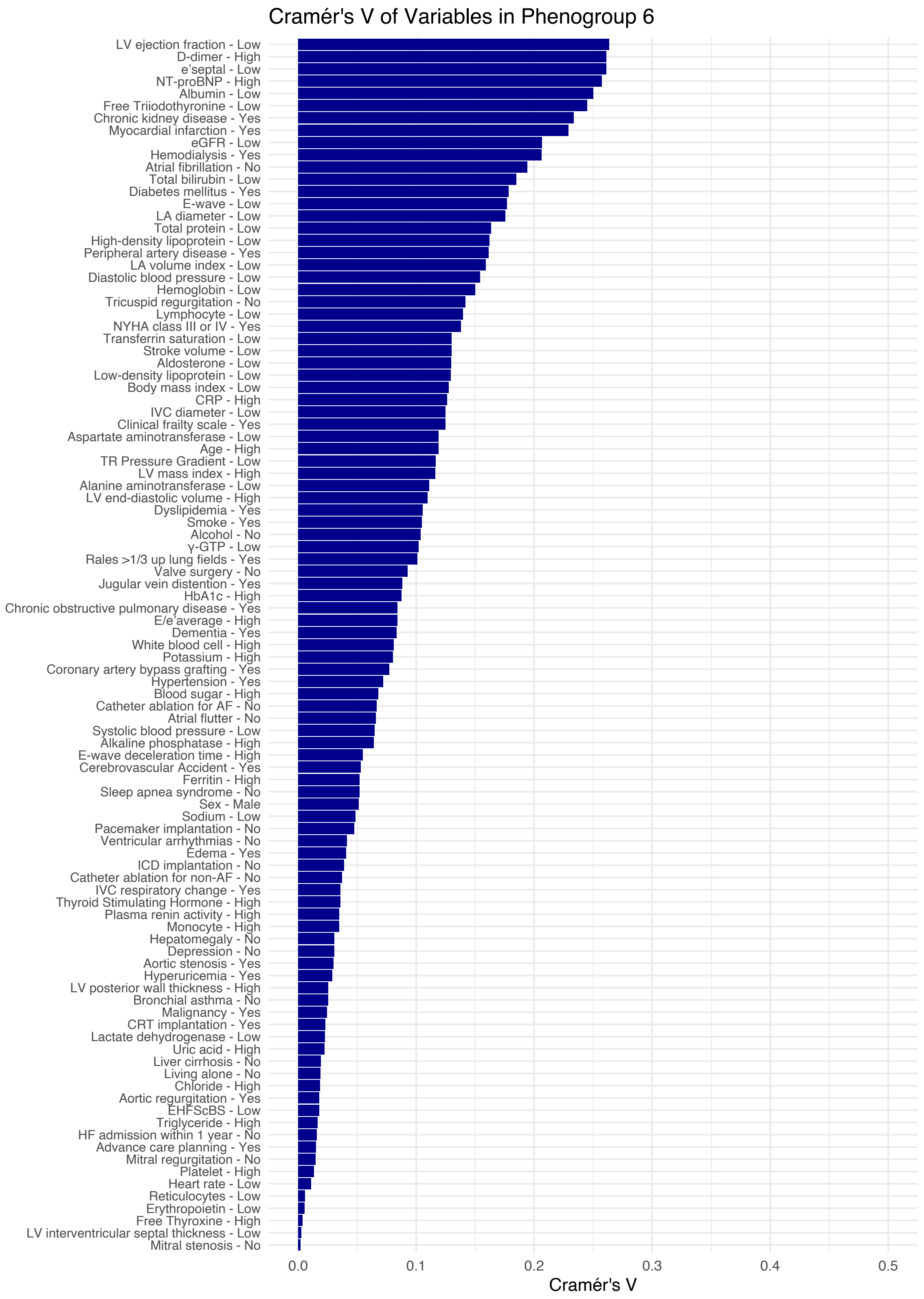

AF, atrial fibrillation; CRT, cardiac resynchronization therapy; CRP, C-reactive protein; EHFScBS, European Heart Failure Self-care Behaviour Scale; eGFR, estimated glomerular filtration rate; γ-GTP, gamma-glutamyl transpeptidase; HbA1c, hemoglobin A1c; HF, heart failure; ICD, implantable cardioverter-defibrillator; IVC, inferior vena cava; LA, left atrium; LV, left ventricle; NT-proBNP, N-terminal pro B-type natriuretic peptide; NYHA, New York Heart Association; TR, tricuspid regurgitation.

Supplementary Figure S10. Feature importance in Phenogroup 7

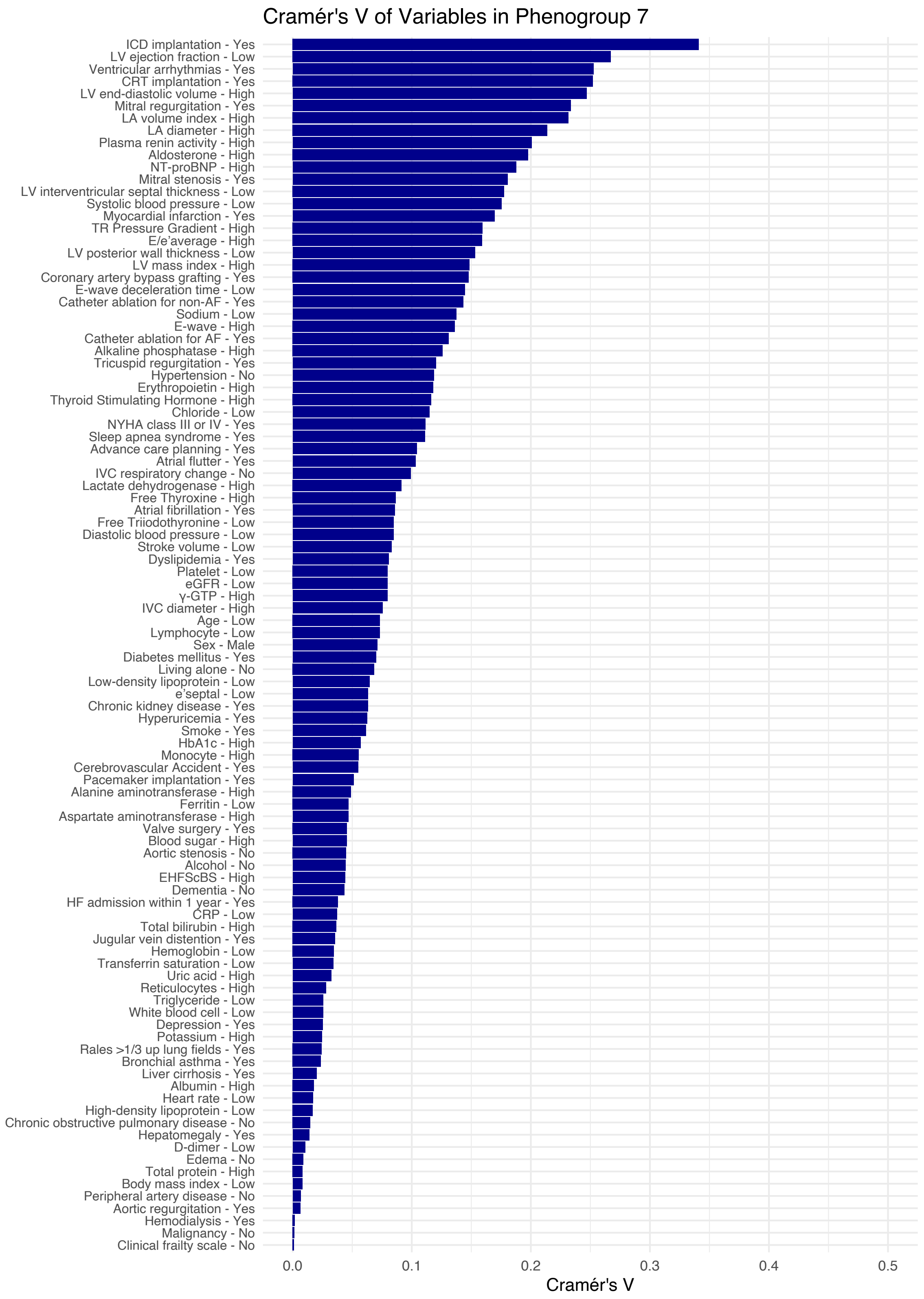

AF, atrial fibrillation; CRT, cardiac resynchronization therapy; CRP, C-reactive protein; EHFScBS, European Heart Failure Self-care Behaviour Scale; eGFR, estimated glomerular filtration rate; γ-GTP, gamma-glutamyl transpeptidase; HbA1c, hemoglobin A1c; HF, heart failure; ICD, implantable cardioverter-defibrillator; IVC, inferior vena cava; LA, left atrium; LV, left ventricle; NT-proBNP, N-terminal pro B-type natriuretic peptide; NYHA, New York Heart Association; TR, tricuspid regurgitation.

Supplementary Figure S11. Feature importance in Phenogroup 8

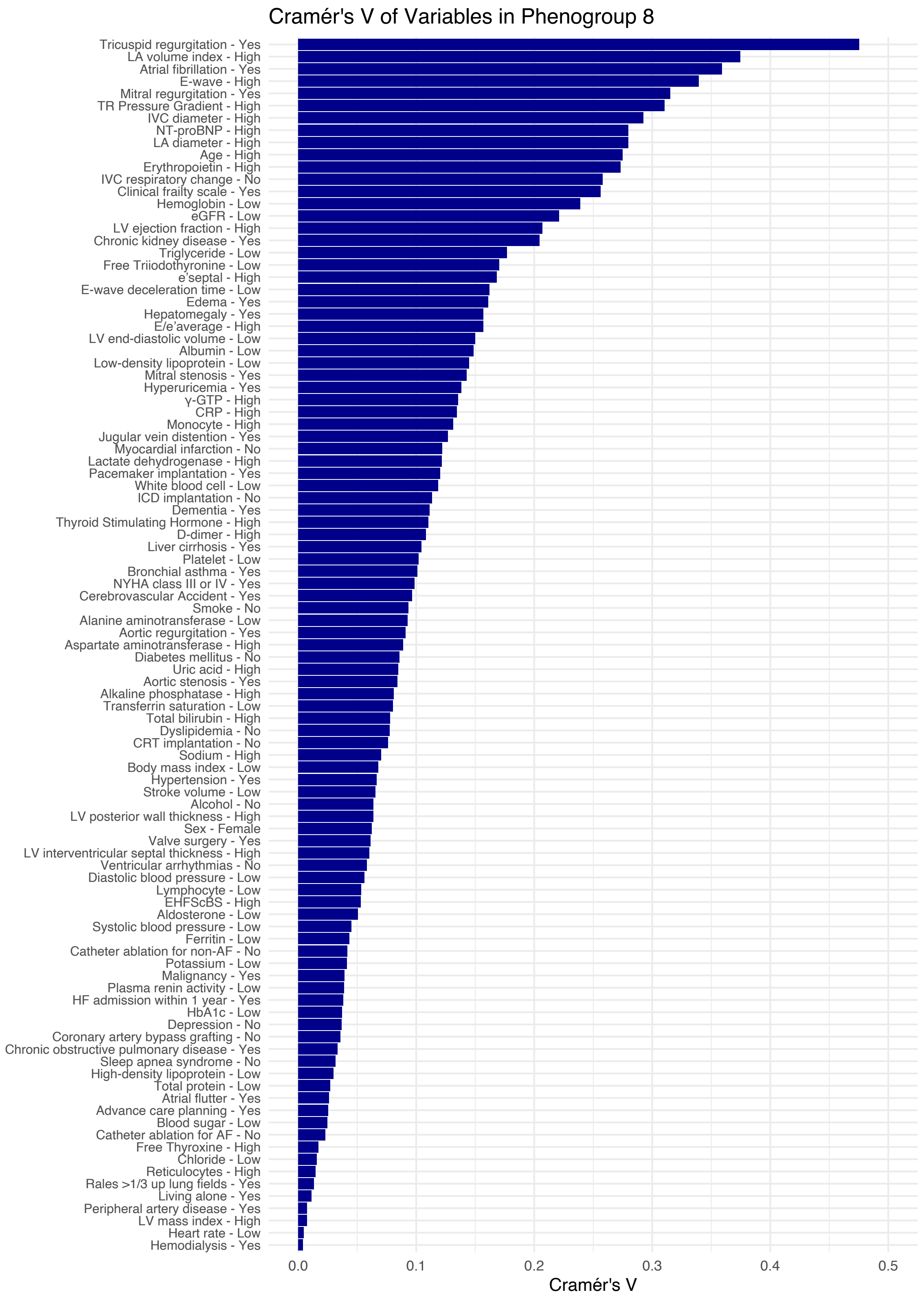

AF, atrial fibrillation; CRT, cardiac resynchronization therapy; CRP, C-reactive protein; EHFScBS, European Heart Failure Self-care Behaviour Scale; eGFR, estimated glomerular filtration rate; γ-GTP, gamma-glutamyl transpeptidase; HbA1c, hemoglobin A1c; HF, heart failure; ICD, implantable cardioverter-defibrillator; IVC, inferior vena cava; LA, left atrium; LV, left ventricle; NT-proBNP, N-terminal pro B-type natriuretic peptide; NYHA, New York Heart Association; TR, tricuspid regurgitation.

Supplementary Figure S12. Sankey diagram showing the association between left ventricular ejection fraction-based classification and eight phenogroups.

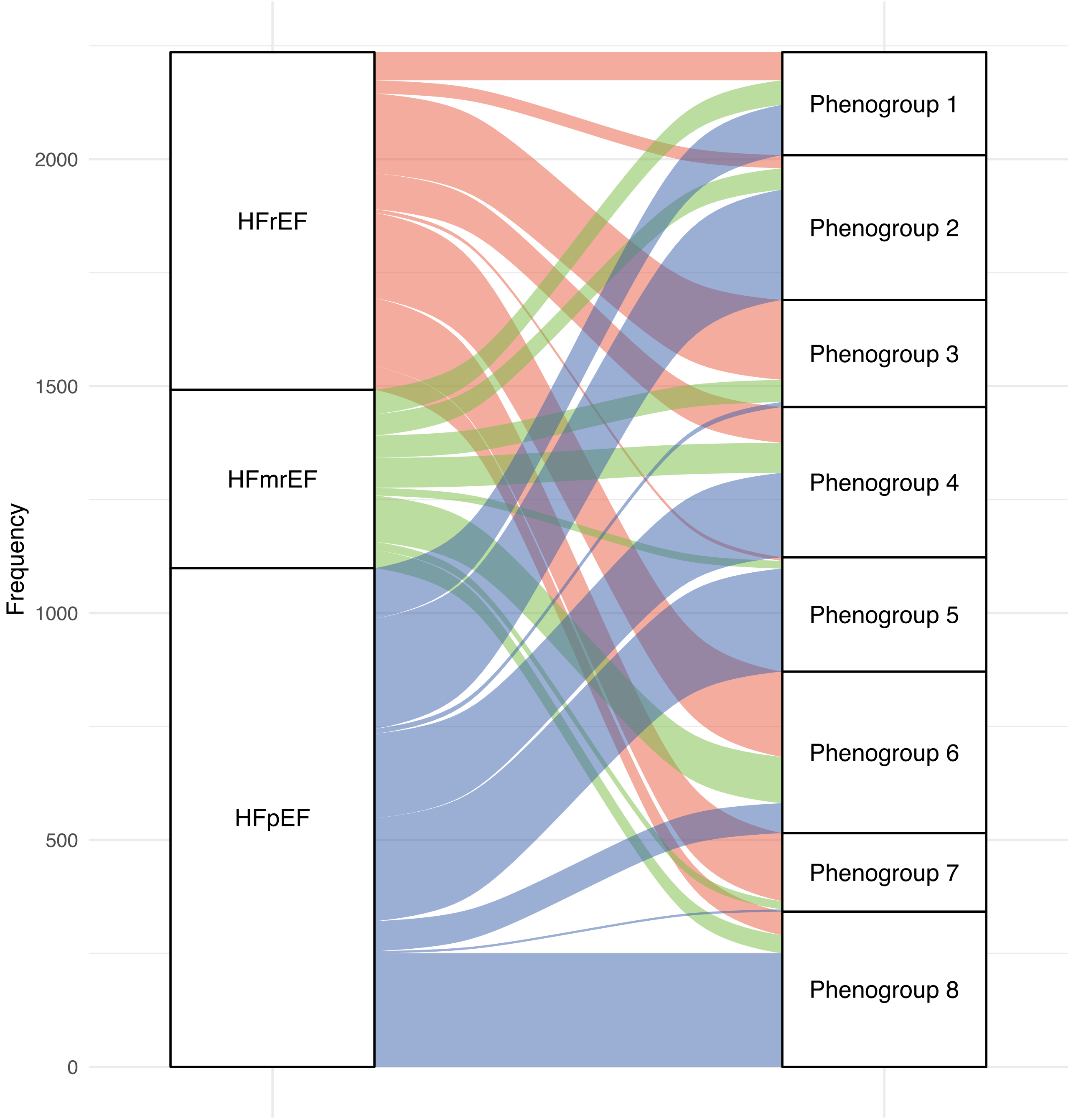

HFrEF, heart failure with reduced ejection fraction; HFmrEF, heart failure with mildly reduced ejection fraction; HFpEF, heart failure with preserved ejection fraction.

Supplementary Figure S13 Log-minus-log survival plots for proportional hazards assumption across phenogroups.

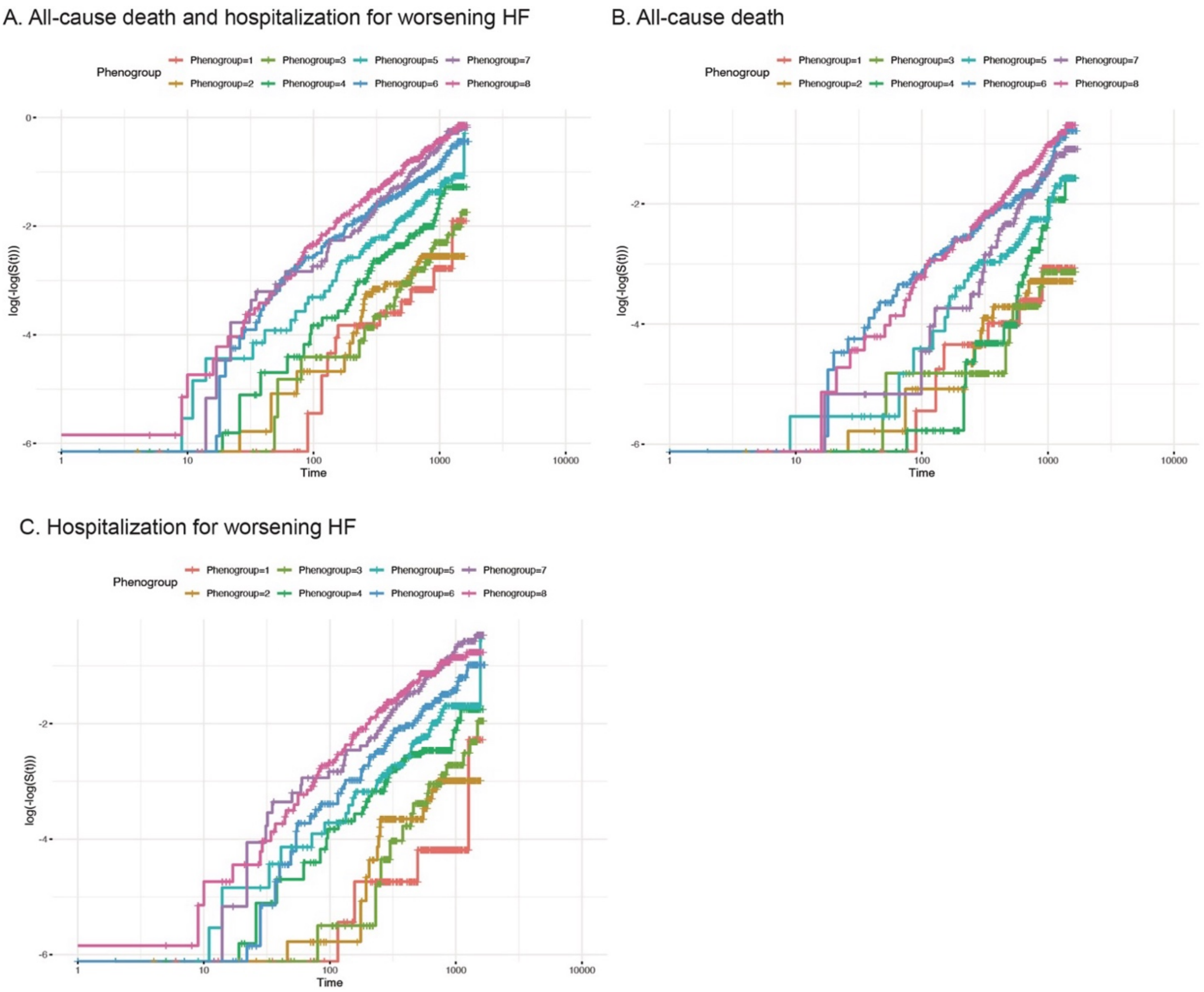

Supplementary Figure S14 Cumulative incidence of all-cause death and hospitalization for worsening heart failure.

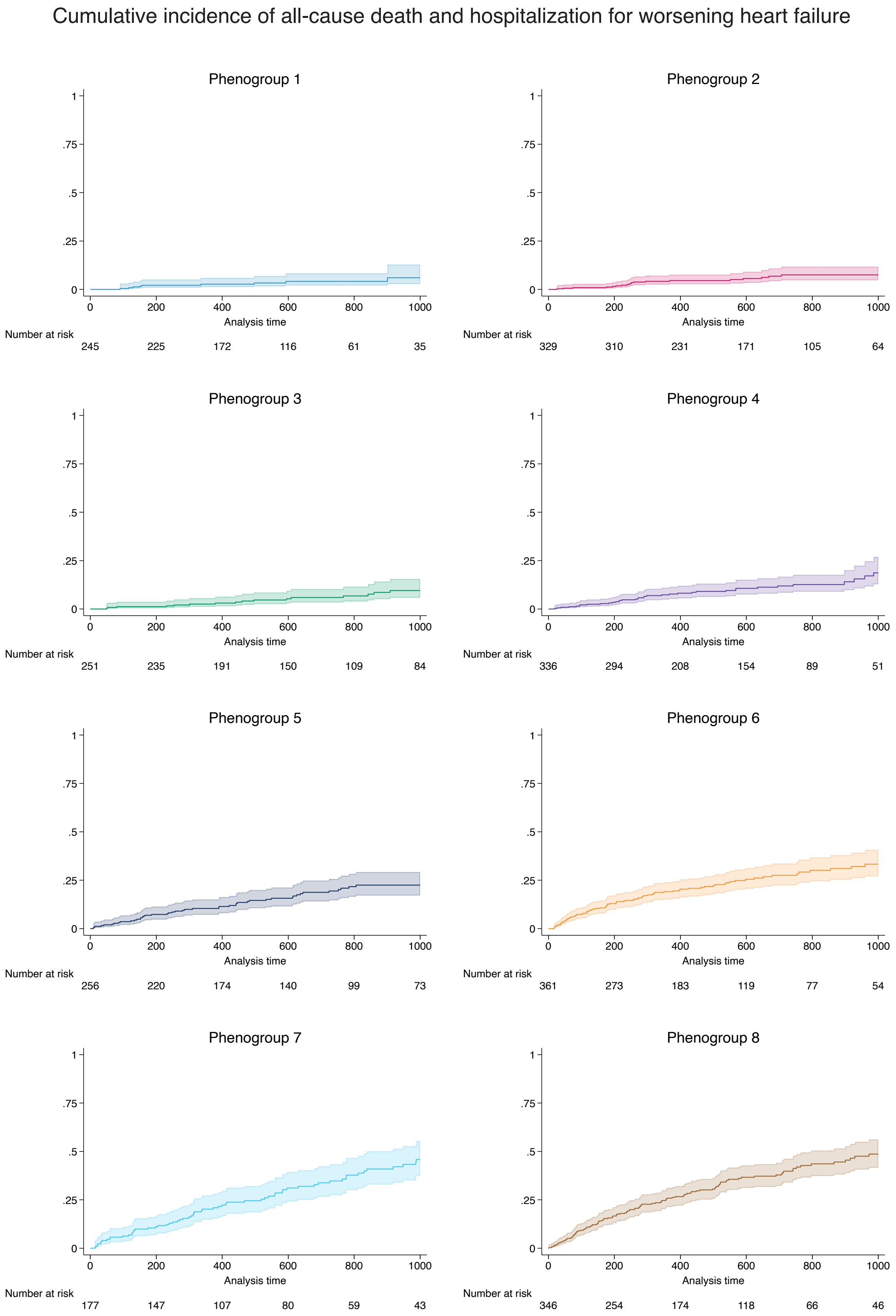

Supplementary Figure S15 Cumulative incidence of all-cause death.

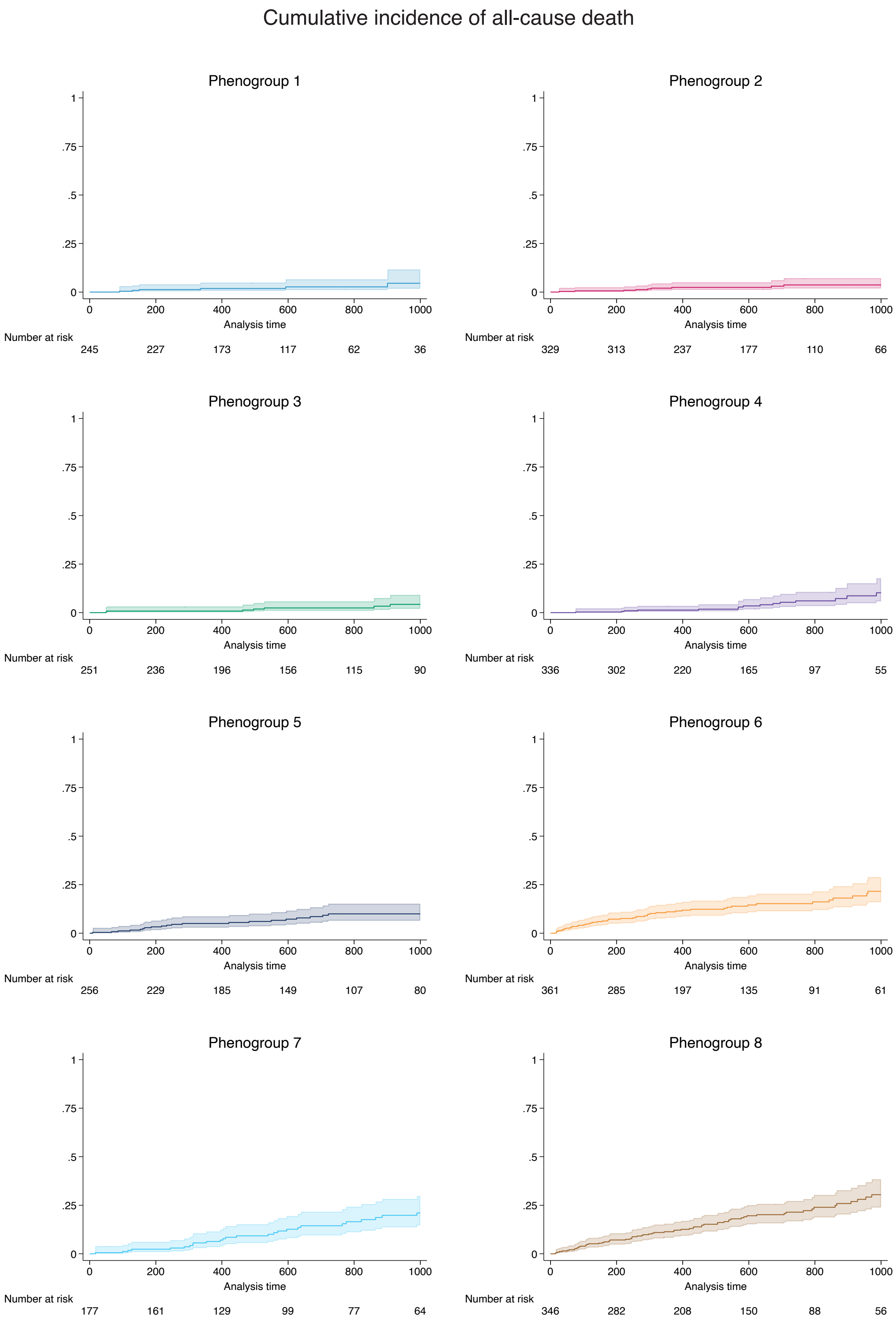

Supplementary Figure S16 Cumulative incidence of hospitalization for worsening heart failure.

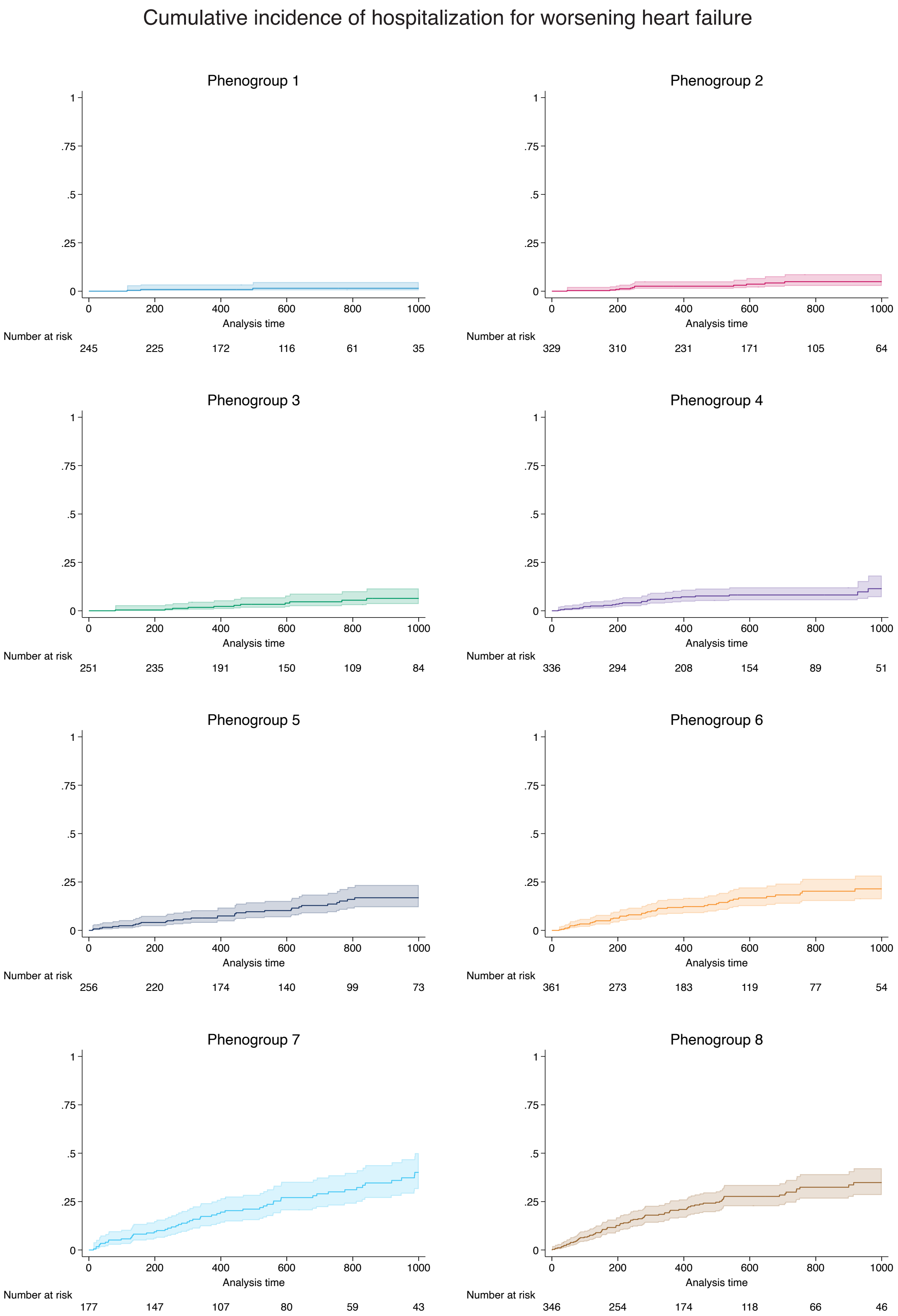

Supplementary Figure S17. Cumulative incidence of worsening HF in Phenogroup 5 patients stratified by atrial fibrillation status. (A) Patients with atrial fibrillation; (B) Patients without atrial fibrillation.

A. Hospitalization for worsening HF in patients with Phenogroup 5 and atrial fibrillation

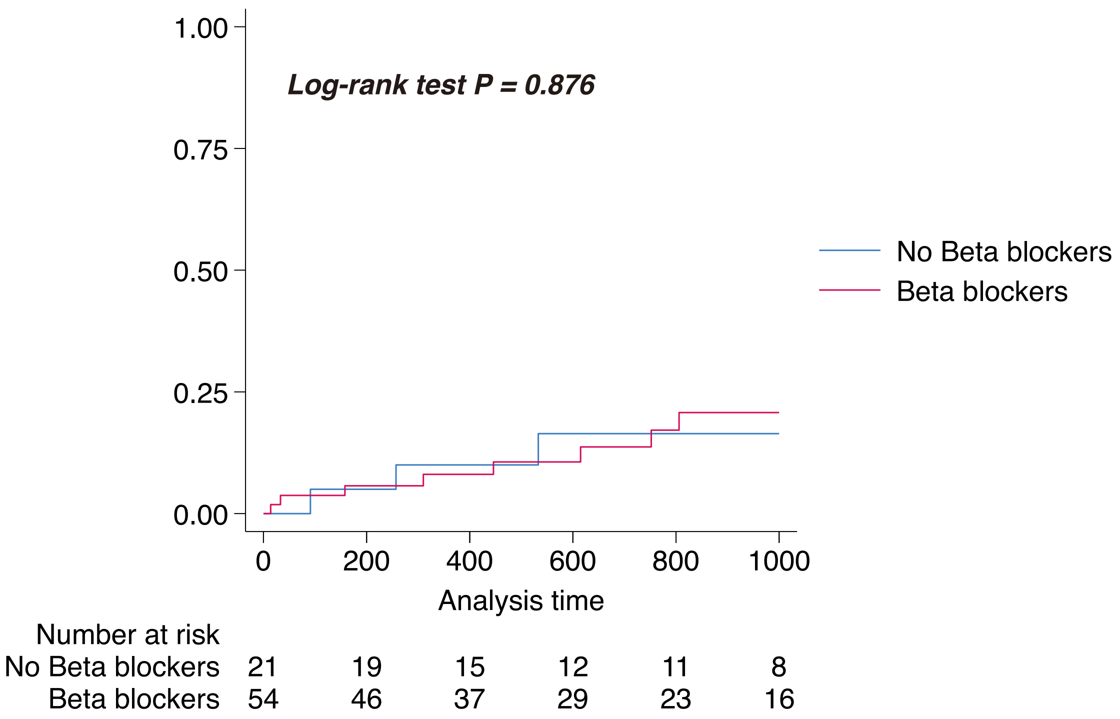

B. Hospitalization for worsening HF in patients with Phenogroup 5 and without atrial fibrillation

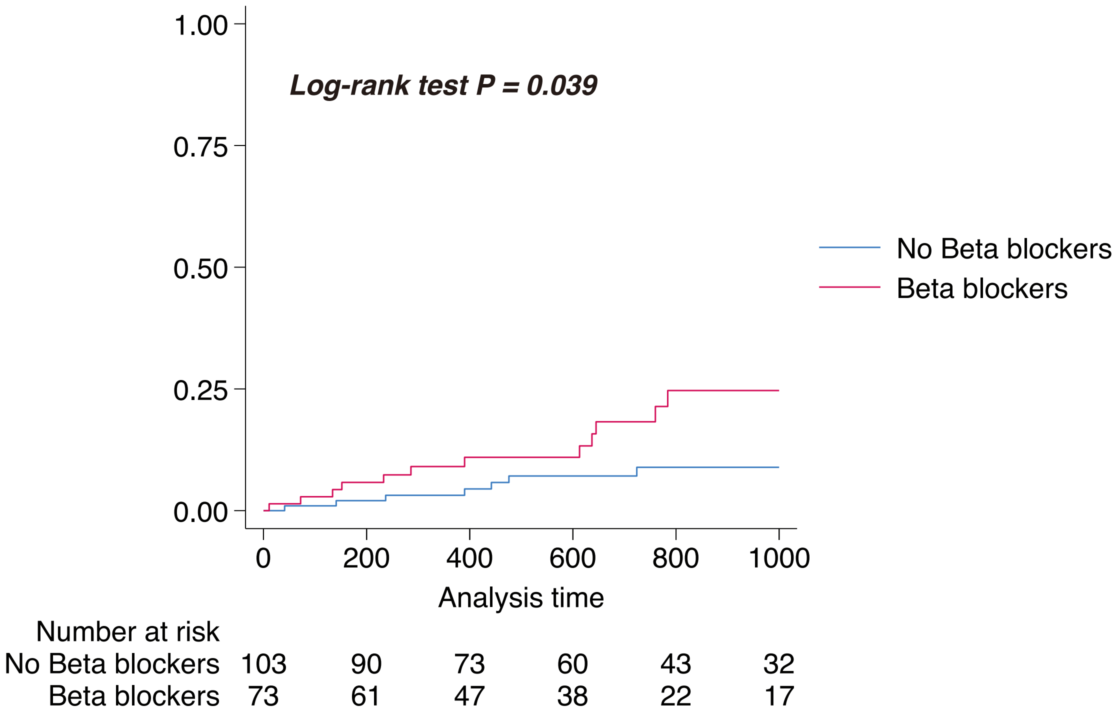

HF, heart failure.

Supplementary Figure S18. Total clustering heatmap of phenogroup concordance between derivation and bootstrap replications

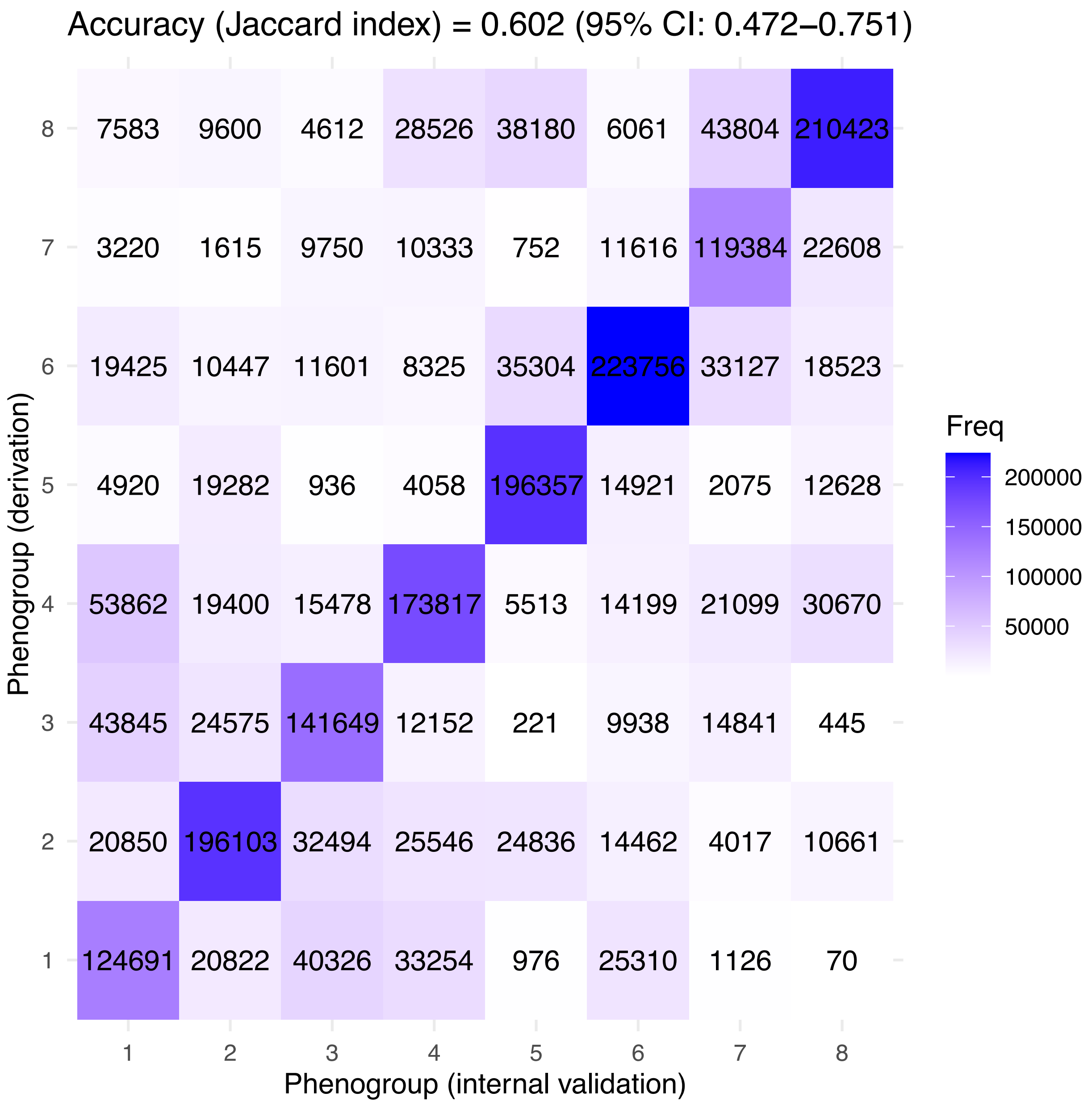

Supplementary Figure S19. Feature importance in Phenogroup 1 based on bootstrap analysis

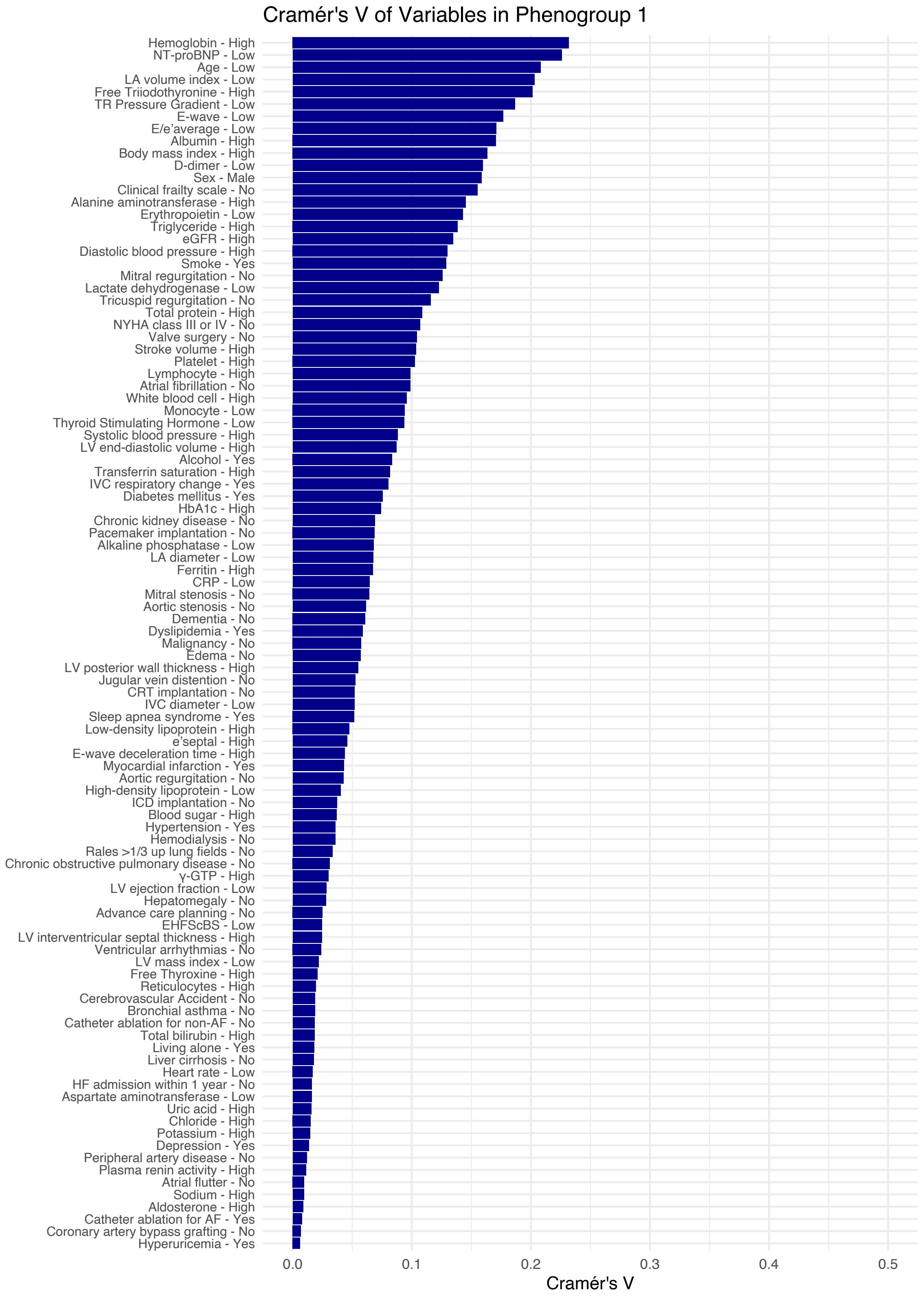

AF, atrial fibrillation; CRT, cardiac resynchronization therapy; CRP, C-reactive protein; EHFScBS, European Heart Failure Self-care Behaviour Scale; eGFR, estimated glomerular filtration rate; γ-GTP, gamma-glutamyl transpeptidase; HbA1c, hemoglobin A1c; HF, heart failure; ICD, implantable cardioverter-defibrillator; IVC, inferior vena cava; LA, left atrium; LV, left ventricle; NT-proBNP, N-terminal pro B-type natriuretic peptide; NYHA, New York Heart Association; TR, tricuspid regurgitation.

Supplementary Figure S20. Feature importance in Phenogroup 2 based on bootstrap analysis

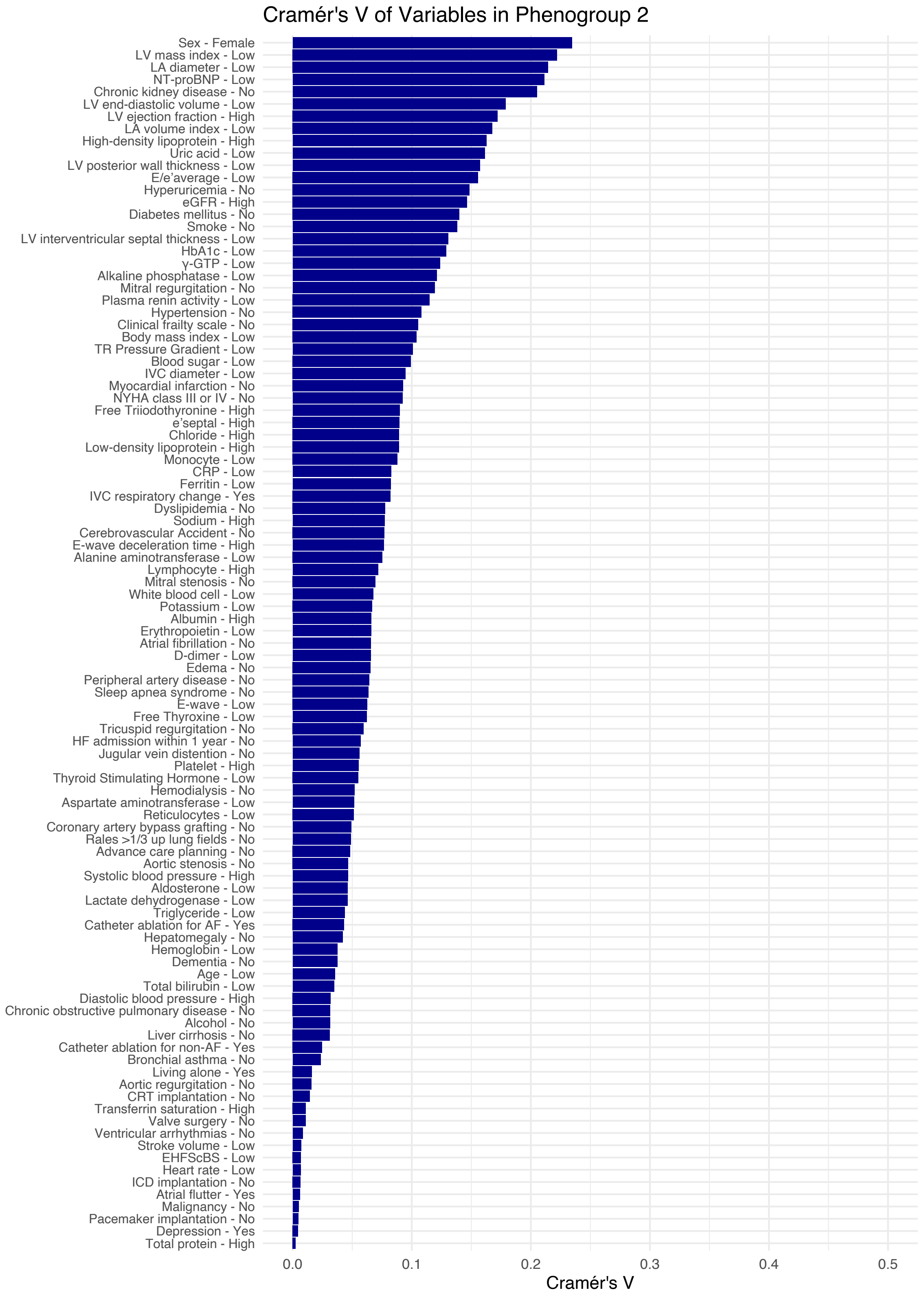

AF, atrial fibrillation; CRT, cardiac resynchronization therapy; CRP, C-reactive protein; EHFScBS, European Heart Failure Self-care Behaviour Scale; eGFR, estimated glomerular filtration rate; γ-GTP, gamma-glutamyl transpeptidase; HbA1c, hemoglobin A1c; HF, heart failure; ICD, implantable cardioverter-defibrillator; IVC, inferior vena cava; LA, left atrium; LV, left ventricle; NT-proBNP, N-terminal pro B-type natriuretic peptide; NYHA, New York Heart Association; TR, tricuspid regurgitation.

Supplementary Figure S21. Feature importance in Phenogroup 3 based on bootstrap analysis

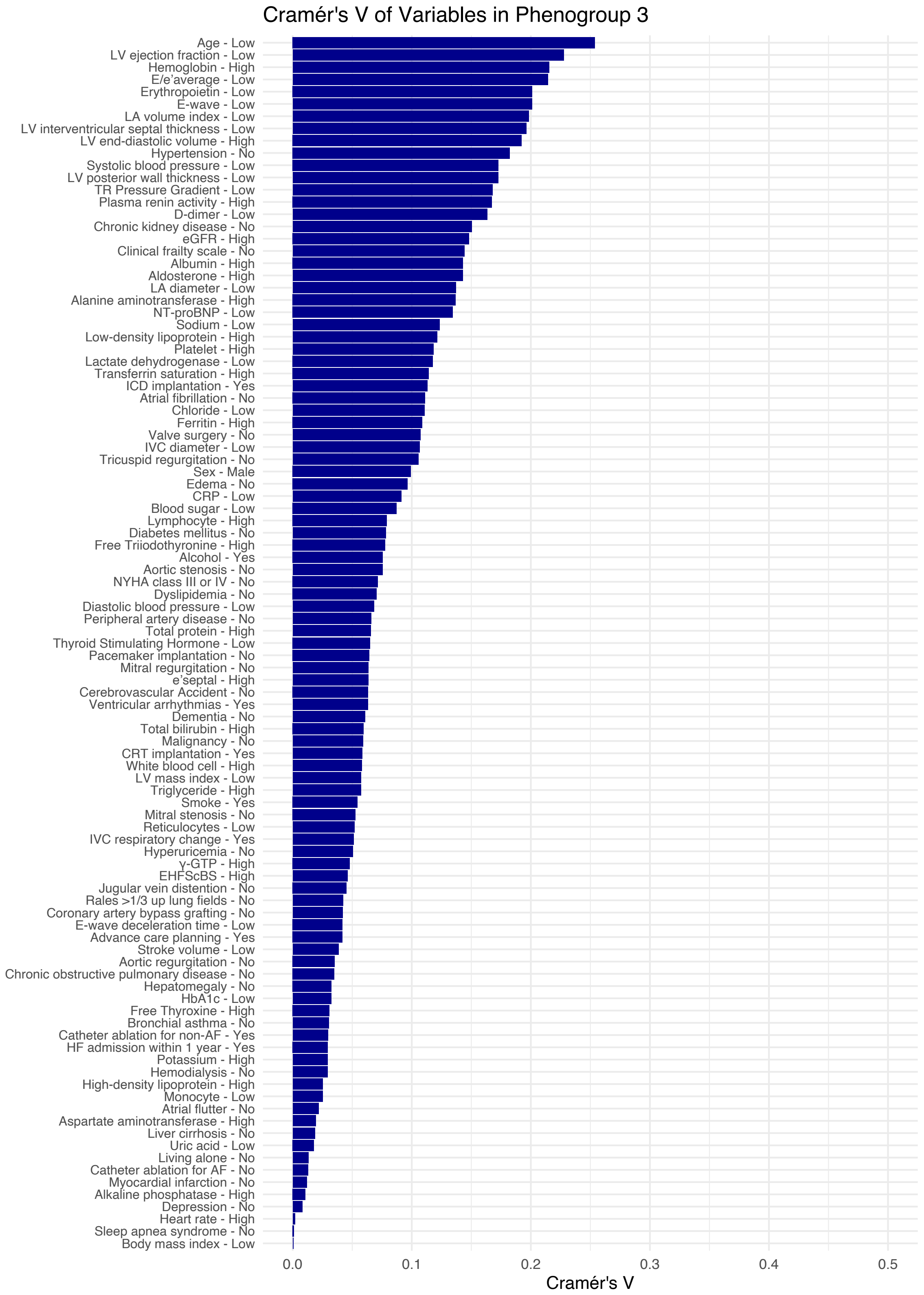

AF, atrial fibrillation; CRT, cardiac resynchronization therapy; CRP, C-reactive protein; EHFScBS, European Heart Failure Self-care Behaviour Scale; eGFR, estimated glomerular filtration rate; γ-GTP, gamma-glutamyl transpeptidase; HbA1c, hemoglobin A1c; HF, heart failure; ICD, implantable cardioverter-defibrillator; IVC, inferior vena cava; LA, left atrium; LV, left ventricle; NT-proBNP, N-terminal pro B-type natriuretic peptide; NYHA, New York Heart Association; TR, tricuspid regurgitation.

Supplementary Figure S22. Feature importance in Phenogroup 4 based on bootstrap analysis

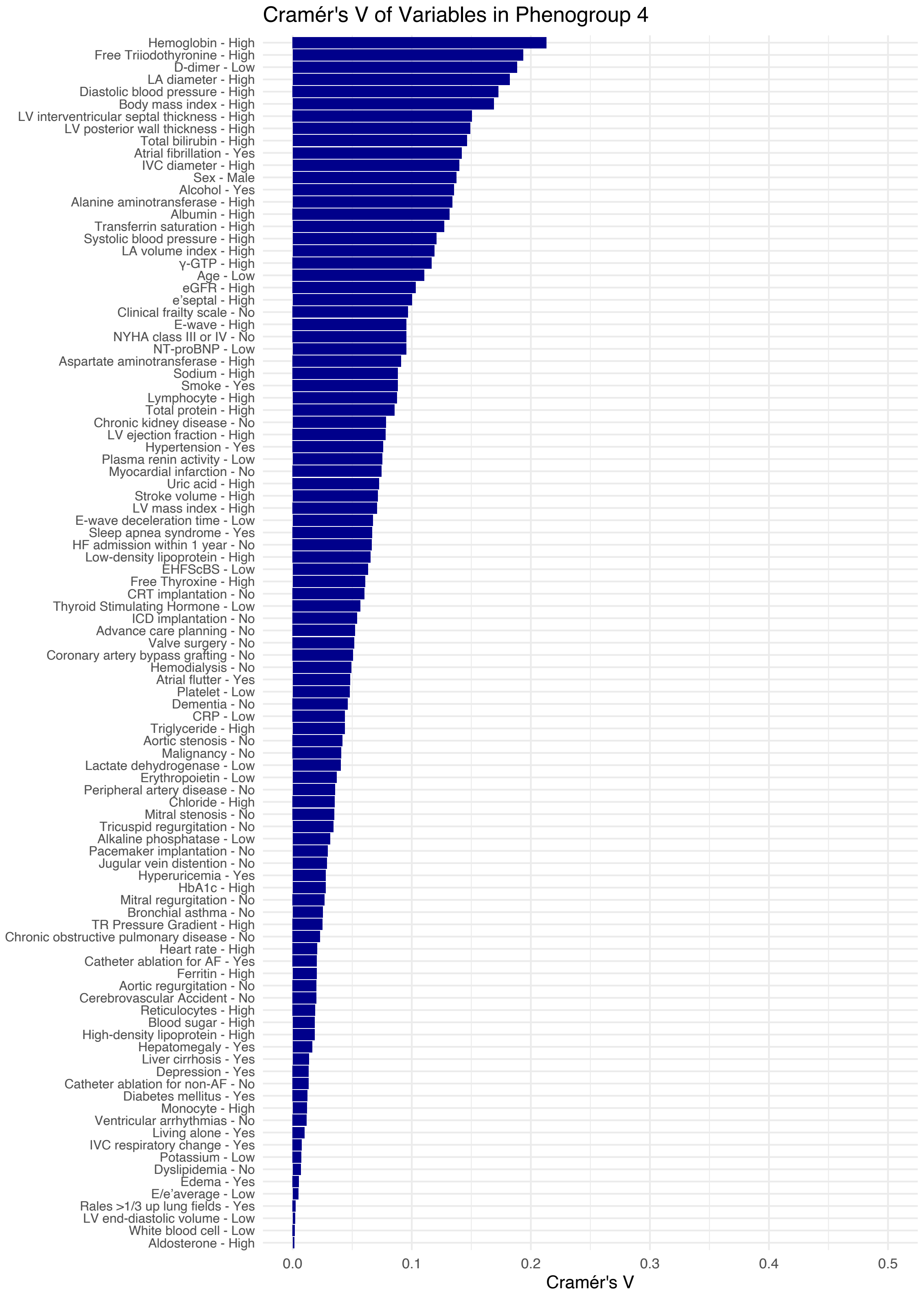

AF, atrial fibrillation; CRT, cardiac resynchronization therapy; CRP, C-reactive protein; EHFScBS, European Heart Failure Self-care Behaviour Scale; eGFR, estimated glomerular filtration rate; γ-GTP, gamma-glutamyl transpeptidase; HbA1c, hemoglobin A1c; HF, heart failure; ICD, implantable cardioverter-defibrillator; IVC, inferior vena cava; LA, left atrium; LV, left ventricle; NT-proBNP, N-terminal pro B-type natriuretic peptide; NYHA, New York Heart Association; TR, tricuspid regurgitation.

Supplementary Figure S23. Feature importance in Phenogroup 5 based on bootstrap analysis

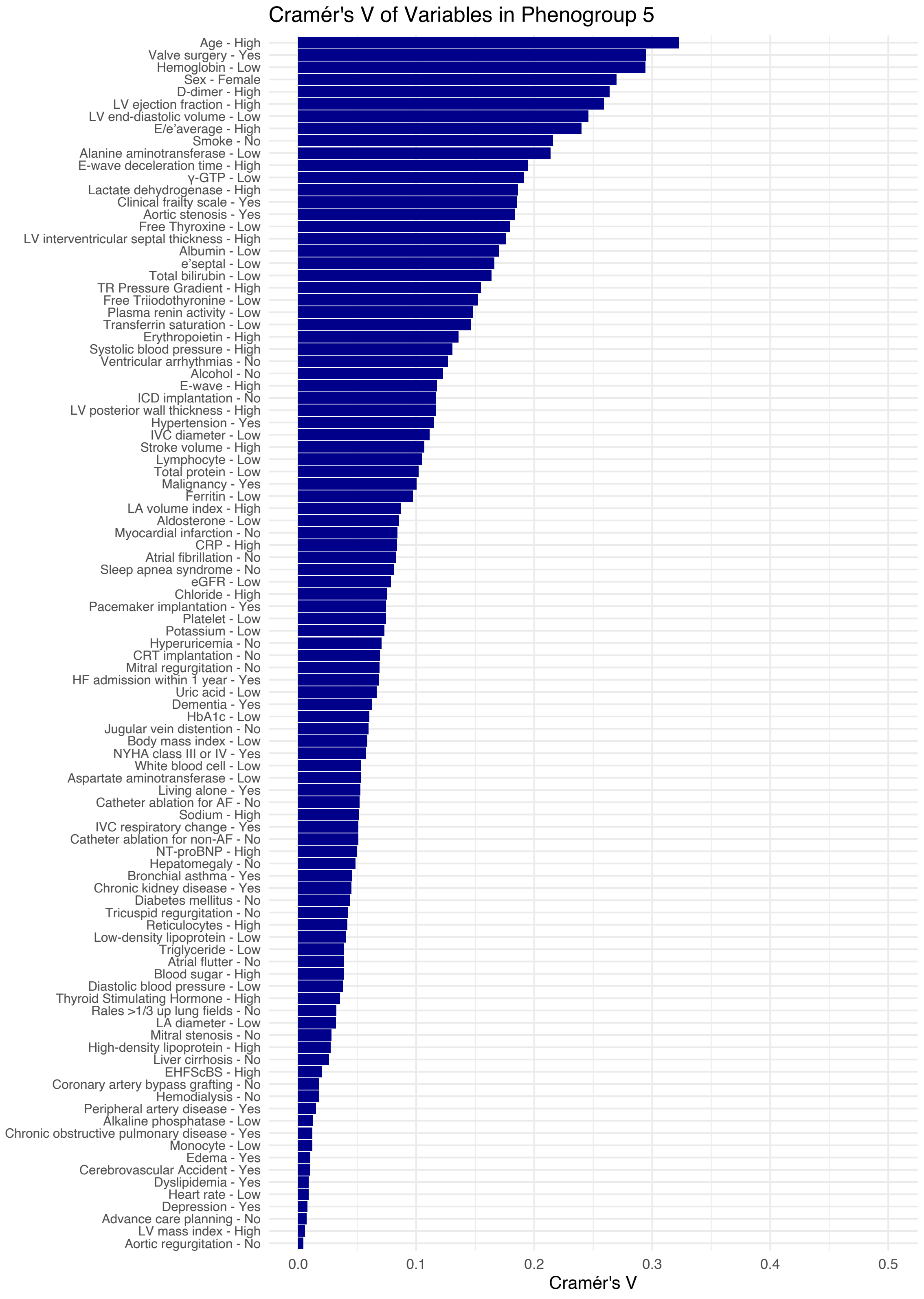

AF, atrial fibrillation; CRT, cardiac resynchronization therapy; CRP, C-reactive protein; EHFScBS, European Heart Failure Self-care Behaviour Scale; eGFR, estimated glomerular filtration rate; γ-GTP, gamma-glutamyl transpeptidase; HbA1c, hemoglobin A1c; HF, heart failure; ICD, implantable cardioverter-defibrillator; IVC, inferior vena cava; LA, left atrium; LV, left ventricle; NT-proBNP, N-terminal pro B-type natriuretic peptide; NYHA, New York Heart Association; TR, tricuspid regurgitation.

Supplementary Figure S24. Feature importance in Phenogroup 6 based on bootstrap analysis

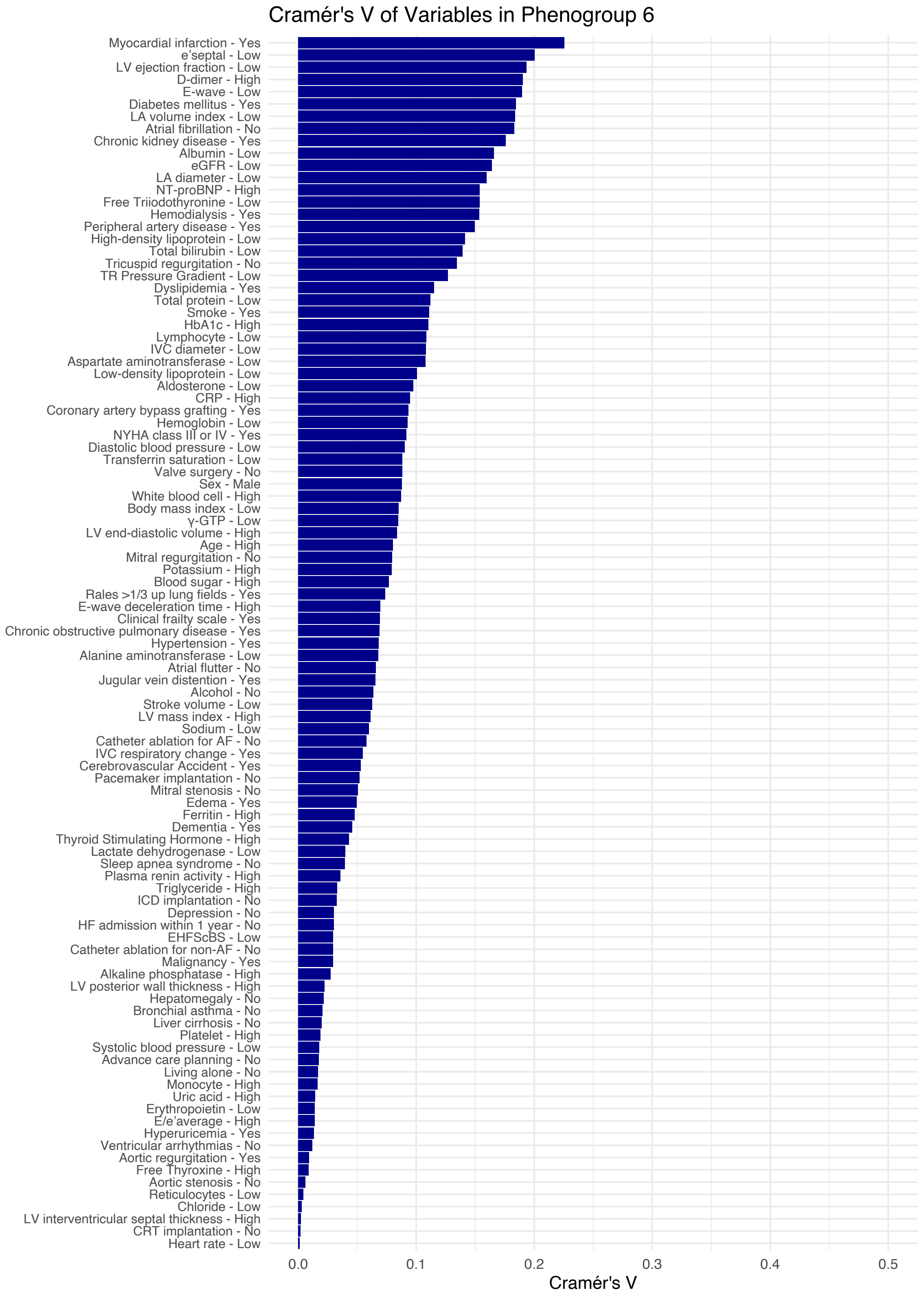

AF, atrial fibrillation; CRT, cardiac resynchronization therapy; CRP, C-reactive protein; EHFScBS, European Heart Failure Self-care Behaviour Scale; eGFR, estimated glomerular filtration rate; γ-GTP, gamma-glutamyl transpeptidase; HbA1c, hemoglobin A1c; HF, heart failure; ICD, implantable cardioverter-defibrillator; IVC, inferior vena cava; LA, left atrium; LV, left ventricle; NT-proBNP, N-terminal pro B-type natriuretic peptide; NYHA, New York Heart Association; TR, tricuspid regurgitation.

Supplementary Figure S25. Feature importance in Phenogroup 7 based on bootstrap analysis

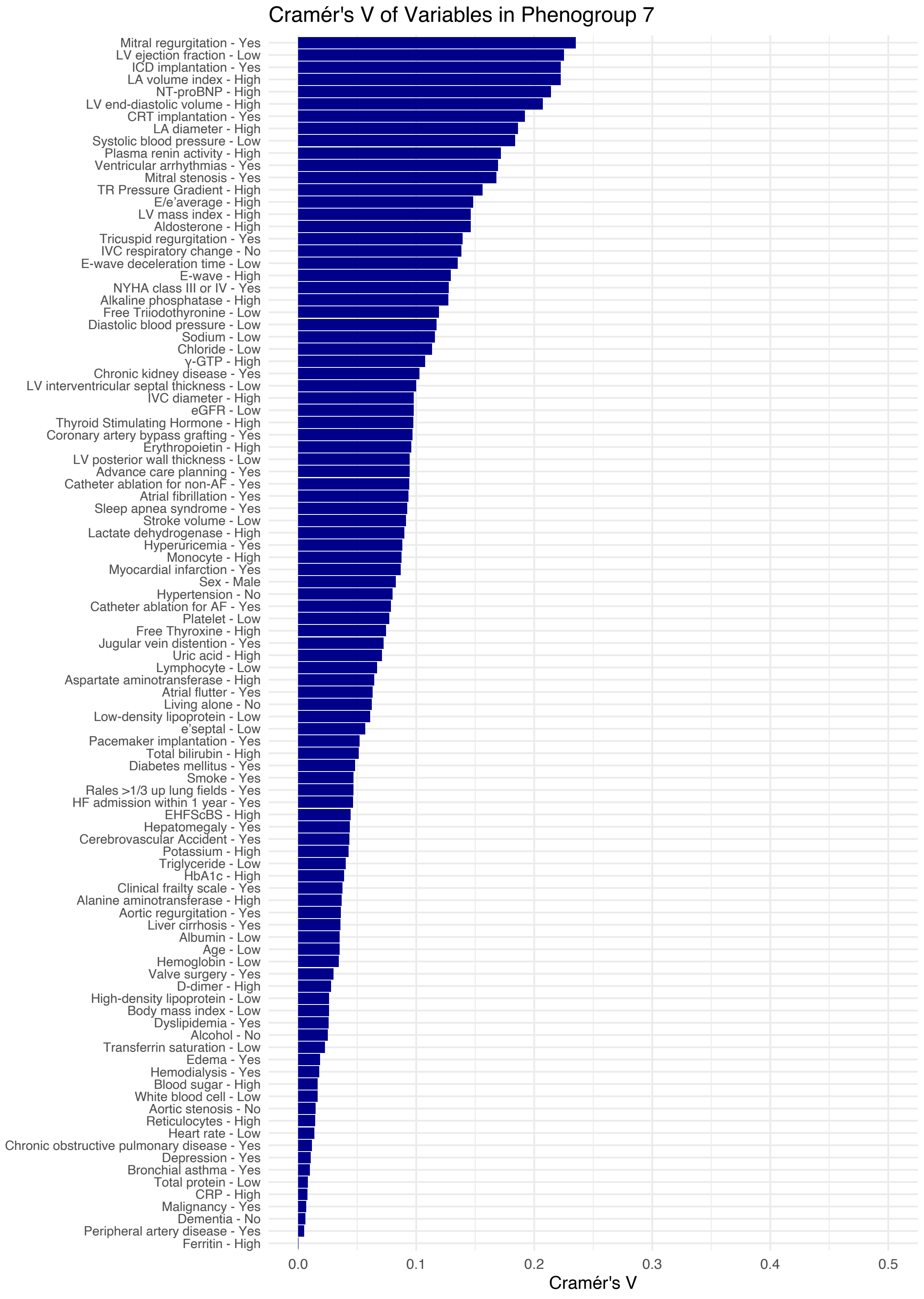

AF, atrial fibrillation; CRT, cardiac resynchronization therapy; CRP, C-reactive protein; EHFScBS, European Heart Failure Self-care Behaviour Scale; eGFR, estimated glomerular filtration rate; γ-GTP, gamma-glutamyl transpeptidase; HbA1c, hemoglobin A1c; HF, heart failure; ICD, implantable cardioverter-defibrillator; IVC, inferior vena cava; LA, left atrium; LV, left ventricle; NT-proBNP, N-terminal pro B-type natriuretic peptide; NYHA, New York Heart Association; TR, tricuspid regurgitation.

Supplementary Figure S26. Feature importance in Phenogroup 8 based on bootstrap analysis

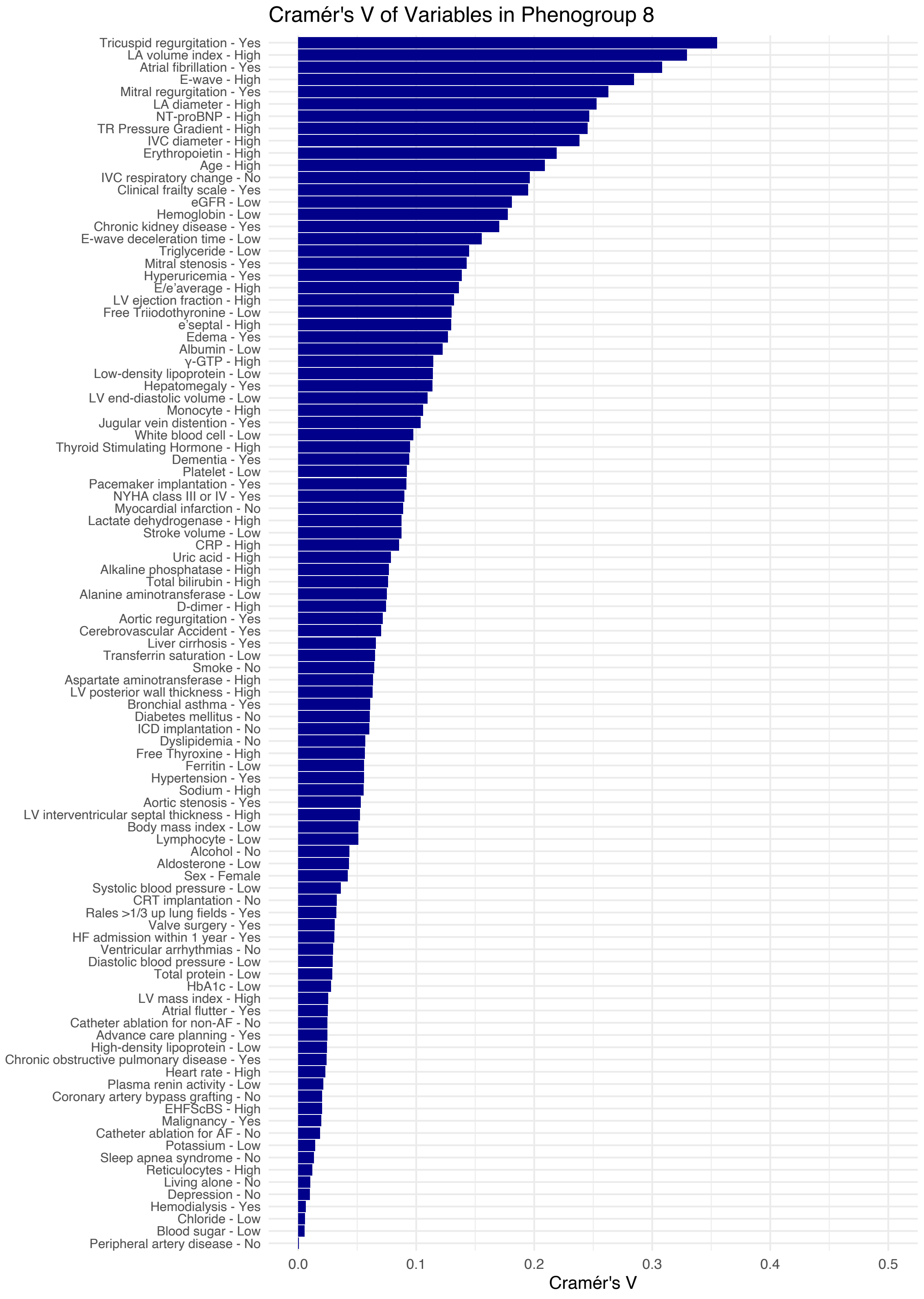

AF, atrial fibrillation; CRT, cardiac resynchronization therapy; CRP, C-reactive protein; EHFScBS, European Heart Failure Self-care Behaviour Scale; eGFR, estimated glomerular filtration rate; γ-GTP, gamma-glutamyl transpeptidase; HbA1c, hemoglobin A1c; HF, heart failure; ICD, implantable cardioverter-defibrillator; IVC, inferior vena cava; LA, left atrium; LV, left ventricle; NT-proBNP, N-terminal pro B-type natriuretic peptide; NYHA, New York Heart Association; TR, tricuspid regurgitation.

Supplementary Figure S27. Cumulative incidence of clinical outcomes among the eight phenogroups identified by bootstrap analysis. (A)All-cause death and HF hospitalization. (B) All-cause death. (C) HF hospitalization

A. All-cause death and hospitalization for worsening HF

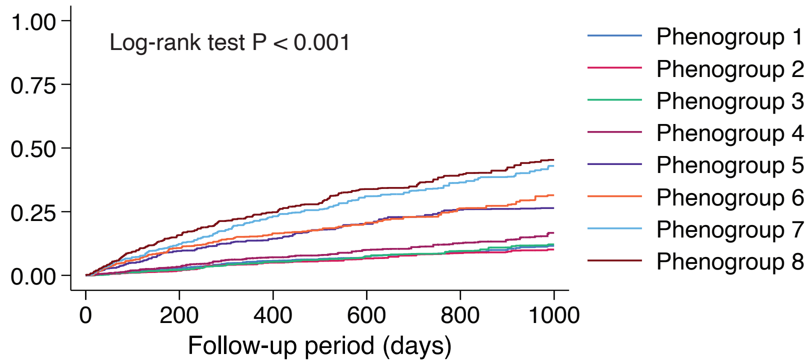

B. All-cause death

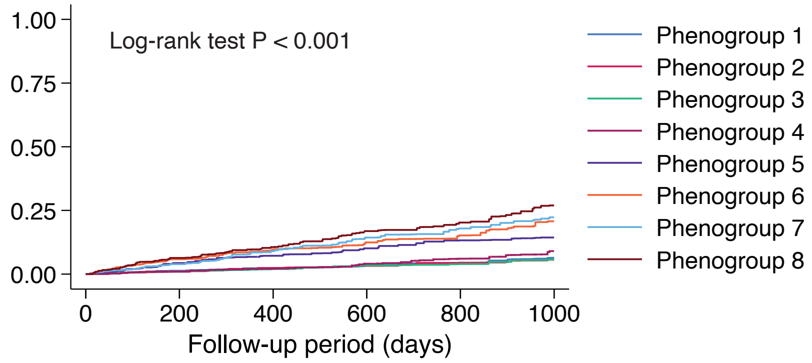

C. Hospitalization for worsening HF

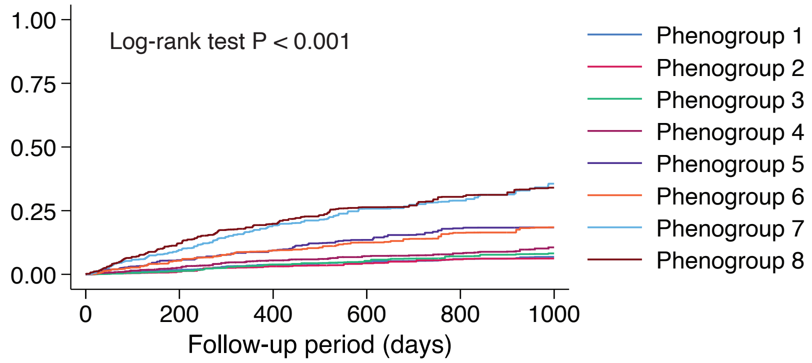

HF, heart failure.

Supplementary Appendix S1. Participating centers and their principal investigators

Participating center	Principal investigator
Department of Cardiovascular Medicine, Faculty of Medicine and Graduate School of Medicine, Hokkaido University, Sapporo, Japan	Toshihisa Anzai
Department of Cardiology, Kushiro City General Hospital, Hokkaido, Japan	Yoshiya Kato
Department of Cardiology, National Hospital Organization Hakodate Medical Center, Hokkaido, Japan	Kazuya Yonezawa
Department of Cardiology, Hakodate Municipal Hospital, Hokkaido, Japan	Yasuhiro Makita
Department of Cardiology, Japan Community Health Care Organization Hokkaido Hospital, Sapporo, Japan	Masashige Takahashi
Department of Cardiology, Keiwakai Ebetsu Hospital, Hokkaido, Japan	Junichi Matsumoto
Department of Cardiology, Hokkaido Chuo Rosai Hospital, Hokkaido, Japan	Ko Motoi
Department of Cardiology, Aishin Memorial Hospital, Sapporo, Japan	Hiroshi Okamoto
Department of Cardiology, Tomakomai City Hospital, Hokkaido, Japan	Masaharu Machida
Department of Cardiology, Japan Red Cross Kitami Hospital, Hokkaido, Japan	Takahiko Saito
Department of Cardiology, Sunagawa City Medical Center, Hokkaido, Japan	Takayuki Hirabayashi
Department of Cardiovascular Medicine, Gunma University Graduate School of Medicine, Gunma, Japan	Masahiko Kurabayashi
Department of Cardiology, Japan Red Cross Date Hospital, Hokkaido, Japan	Shigeru Takechi
Department of Cardiology, Tonan Hospital, Hokkaido, Japan	Yutaka Matsui
Department of Cardiology, Obihiro Kyokai Hospital, Hokkaido, Japan	Ichiro Yoshida
Department of Cardiology, Otaru Kyokai Hospital, Hokkaido, Japan	Shigeo Kakinoki
Department of Cardiology, Hokkaido P.W.F.A.C. Sapporo-Kosei General Hospital, Sapporo, Japan	Yasumi Igarashi
Department of Cardiology, National Hospital Organization Hokkaido Medical Center, Sapporo, Japan	Takashi Takenaka
Department of Cardiology, Japan Self Defense Forces Hospital Sapporo, Sapporo, Japan	Akinori Takahashi
Department of Cardiology, Sapporo Makomanai Hospital, Sapporo, Japan	Eiichiro Imamura
Department of Cardiology, Cardiovascular Center Tokeidai Memorial Hospital, Sapporo, Japan	Kazushi Urasawa

Department of Cardiology, Caress Sapporo Hokko Memorial Clinic, Sapporo, Japan	Ichiro Sakuma
Department of Cardiology, Caress Sapporo Hokko Memorial Hospital, Sapporo, Japan	Masayuki Sakurai
Department of Cardiovascular Medicine, NTT Medical Center Sapporo, Sapporo, Japan	Kazuyuki Noriyasu
Department of Cardiology, Sapporo City General Hospital, Sapporo, Japan	Hisashi Yokoshiki
Department of Cardiology, Sapporo Kojinkai Memorial Hospital, Sapporo, Japan	Takehiro Yamashita
Department of Cardiology, Tokyo Medical University Hospital, Tokyo, Japan	Hiroki Nakano
